# Autophagy regulates sex steroid hormone synthesis through lysosomal degradation of lipid droplets in human ovary and testis

**DOI:** 10.1038/s41419-023-05864-3

**Published:** 2023-05-26

**Authors:** Yashar Esmaeilian, Francesko Hela, Gamze Bildik, Ece İltumur, Sevgi Yusufoglu, Ceren Sultan Yildiz, Kayhan Yakin, Yakup Kordan, Ozgur Oktem

**Affiliations:** 1 Research Center for Translational Medicine, Koç University, Istanbul, 34450 Turkey; 2 The Graduate School of Health Sciences, Koç University, Istanbul, 34450 Turkey; 3 Department of Experimental Therapeutics, the University of Texas MD Anderson Cancer Center, Houston, TX 77030 USA; 4 Department of Obstetrics and Gynecology, Koç University School of Medicine, Istanbul, Turkey; 5 Department of Urology, Koç University School of Medicine, Istanbul, Turkey

**Keywords:** Macroautophagy, Endocrine reproductive disorders

## Abstract

Autophagy is an evolutionarily conserved process that aims to maintain the energy homeostasis of the cell by recycling long-lived proteins and organelles. Previous studies documented the role of autophagy in sex steroid hormone biosynthesis in different animal models and human testis. Here we demonstrate in this study that sex steroid hormones estrogen and progesterone are produced through the same autophagy-mediated mechanism in the human ovary in addition to the human testis. In brief, pharmacological inhibition and genetic interruption of autophagy through silencing of autophagy genes (Beclin1 and ATG5) via siRNA and shRNA technologies significantly reduced basal and gonadotropin-stimulated estradiol (E_2_), progesterone (P_4_) and testosterone (T) production in the ex vivo explant tissue culture of ovary and testis and primary and immortalized granulosa cells. Consistent with the findings of the previous works, we observed that lipophagy, a special form of autophagy, mediates the association of the lipid droplets (LD)s with lysosome to deliver the lipid cargo within the LDs to lysosomes for degradation in order to release free cholesterol required for steroid synthesis. Gonadotropin hormones are likely to augment the production of sex steroid hormones by upregulating the expression of autophagy genes, accelerating autophagic flux and promoting the association of LDs with autophagosome and lysosome. Moreover, we detected some aberrations at different steps of lipophagy-mediated P_4_ production in the luteinized GCs of women with defective ovarian luteal function. The progression of autophagy and the fusion of the LDs with lysosome are markedly defective, along with reduced P_4_ production in these patients. Our data, together with the findings of the previous works, may have significant clinical implications by opening a new avenue in understanding and treatment of a wide range of diseases, from reproductive disorders to sex steroid-producing neoplasms, sex steroid-dependent malignancies (breast, endometrium, prostate) and benign disorders (endometriosis).

## Introduction

Steroidogenesis, the biosynthesis of all steroid hormones, including sex steroids (androgens, estrogens and progesterone), starts from a common precursor molecule, cholesterol, and involves the complex biosynthetic pathways in mitochondria [1]. Most of the steroidogenic cholesterol is derived from the circulating low-density lipoproteins (LDLs), which are internalized by receptor-mediated endocytosis after binding to LDL receptors. The resultant endocytic vesicles fuse with lysosome, and engulfed LDL proteins are degraded by proteolysis and release the cholesterol esters, which are subsequently hydrolyzed to free cholesterol by lysosomal acid lipase (LAL) [1]. In steroidogenic cells, free cholesterol is either transported to mitochondria via a carrier protein StAR (Steroidogenic acute regulatory protein) or stored in the LDs following re-esterification by acyl-coenzyme-A-cholesterol-acyl-transferase (ACAT), also known as sterol-O-acetyltransferase (SOAT1) [1]. The intracellular trafficking of cholesterol for steroid hormone production has been the subject of extensive research for the last 50 years and has not been fully elucidated. The autophagic process is an evolutionarily conserved process that aims to maintain the energy homeostasis of the cell by recycling long-lived proteins and organelles [2]. It was also shown that autophagy also regulates lipid metabolism through the lysosomal degradation of lipids, a special form of autophagic process called lipophagy [3]. Previous pioneering studies demonstrated in different animal models and human testis that lipophagy plays a pivotal role in sex steroid hormone biosynthesis by mediating lysosomal degradation of lipid droplets (LDs) to release cholesterol for steroid hormone synthesis [4–8].

In this paper, we aimed (1) to analyze the role of lipophagy in basal and gonadotropin-stimulated estrogen, progesterone and androgen biosynthesis in human gonads using ex vivo tissue and in vitro cell culture models and (2) to investigate if there is any perturbation in sex steroid production through this mechanism in patients with defective ovarian luteal function, which is characterized by compromised progesterone production and low pregnancy and high miscarriage rates [9–12].

## Material and methods

This study was approved by the institutional review board of Koç University (IRB# 2017.141.IRB2.069 and IRB#2019.299.IRB2.092).

Experimental methodologies used in the study have been described in detail in Supplementary Data.

### Patients

The demographic characteristics of the patients are summarized in Supplementary Table S1. Primary luteinized granulosa cells (GCs) were obtained from follicular aspirates of the patients (*n* = 95) during the oocyte retrieval procedure as described previously [9, 13–15]. Ovarian stimulation with gonadotropin hormones was performed with the same protocol (recombinant FSH and gonadotropin-releasing hormone antagonist) in all patients. Ovulation was triggered either conventionally with recombinant hCG (Ovitrelle) if the ovarian response to gonadotropin was normal or; with a GnRH analog (Leuprolide acetate) when there is a hyper-response to ovarian stimulation. Oocyte retrieval was performed 36 h after the ovulation trigger. Recovered luteinized GCs were processed and analyzed separately for each patient. Corpus luteum tissue samples (*n* = 5) were obtained from the patients undergoing laparoscopic ovarian surgeries for benign ovarian cysts at the early luteal phase of their menstrual cycle. Testicular tissue samples (*n* = 5) were isolated from the patients undergoing orchiectomy operations. All patients gave informed consent for their tissue/cells to be used in the experiments.

### Preparation of the gonadal tissue samples and the cells

Patient-derived ovarian corpus luteum (CL) tissue samples, primary luteinized granulosa cells, and immortalized mitotic non-luteinized granulosa cell line (HGrC1) were used to study the role of autophagy in progesterone (P_4_) and estrogen (estradiol:E_2_) synthesis arms of steroidogenesis, respectively. CL and luteinized GCs are frequently used by our group and other researchers to study ovarian biology, steroidogenesis and luteal phase characteristics of IVF cycles [9, 13, 15–19]. To investigate the role of autophagy in estrogen synthesis of the steroidogenic pathway, we utilized mitotic human granulosa cell line HGrC1, which is a non-luteinized granulosa cell line expressing enzymes related to steroidogenesis, such as steroidogenic acute regulatory protein (StAR), aromatase and gonadotropin receptors. These cells are not capable of undergoing luteinization, resembling the characteristics of granulosa cells belonging to follicles in the early stage. HGrC1 might also be capable of displaying the growth transition from a gonadotropin-independent status to a gonadotropin-dependent one [20]. These cells proliferate, express aromatase enzyme and solely produce E_2_ hormone at baseline and in response to FSH in testosterone-supplemented culture media, but do not express LH receptor and have no ability to undergo luteinization and P_4_ production [14, 21, 22]. This cell line was a gift from Dr. Ikara Iwase (Nagoya University, Japan). Primary and immortalized granulosa cells were checked regularly to make sure that they were free of mycoplasma contamination. To study the role of autophagy in testosterone production, human testicular tissue samples were used. Ovarian and testicular tissue samples were immediately transferred in 50-ml falcon tubes in HEPES-buffered DMEM-F12 media supplemented with 10% FBS to the sterile culture hood. Then, the samples were cut into 0.5 × 0.5 cm cubes and cultured in 6-well culture plates using 3 ml complete culture media (DMEM-F12 + 10%FBS) per well with and without indicated chemicals or drugs for 24 h. All tissue samples and cells were cultured using DMEM-F12 culture medium supplemented with 10% fetal bovine serum at 37 °C and 5% CO_2_ as described previously [9, 23]. Recombinant hormones (hCG, LH and FSH) and pharmacological autophagy inhibitors (chloroquine and vinblastine) were added to the culture media at indicated concentrations.

### Chemicals

Recombinant forms of human chorionic gonadotropin (Ovitrelle), luteinizing hormone (LH, Luveris) and follicle-stimulating hormone (FSH, Gonal-F) were purchased from Merck Serono (Merck Serono, Istanbul, Turkiye). Testosterone (cat no: C-IIIN 86500), rapamycin (cat no: 553211) and chloroquine (Cas no:50-63-5) were from Merck Millipore Sigma (Istanbul, Turkiye).

### Viability assay

A live/dead cell assay was performed with 1 μM YO-PRO-1 (ThermoFisher), a green fluorescent carbocyanine nucleic acid stain absorbed by only apoptotic cells, whereas live cells are impermeable to it. Hoechst 33342 (1 μg/ml, ThermoFisher) was used for counterstaining. Live/dead cell imaging of the cells was undertaken under appropriate channels using a fluorescence microscope (IX71; Olympus, Japan).

### Gene expression analysis

RNA isolation was performed with the Quick-RNA MicroPrep Kit (Zymo Research, Irvine, CA, USA) according to the manufacturer’s instructions. RNA was quantified with a spectrophotometric read at 260 nm by Nanodrop 2000 (ThermoFisher Scientific, MA, USA), and 500 ng cDNA was prepared by using M-MLV Reverse Transcriptase (Invitrogen). Quantitative real-time expressions of mRNAs of interest were detected and compared by using the Light Cycler 480 SYBR Green I Master (Roche, Germany). The primers used in the study are shown in Supplementary Table S2. The means and SDs were calculated from three different readouts taken for each target gene in the qRT-PCR assay. We have used the ΔΔCt method for the relative quantitation of target genes [9, 21, 23, 24].

### siRNA transfection

The cells were transfected following the Lipofectamine^TM^ 3000 Reagent (ThermoFisher), Beclin1 siRNA and Atg5 siRNA (Qiagen). Briefly, Lipofectamin3000 and siRNA complexes were prepared and added to the wells for 24 h incubation. Regarding the evaluation of the effect of hCG stimulation on the steroidogenesis activity of siRNA-treated cells, hCG was added at 24 h post-transfection period to the new complete culture media at a concentration of 10 IU/ml.

### shRNA transduction

Production of plasmids and viral supernatants is illustrated in Supplementary Information. HGrC1 cells were transduced using Beclin1 shRNA and FF shRNA (2 µg/ml) in the presence of 5 µg/ml protamine sulfate (Sigma) for two consecutive days. Transduced cells were selected with 2 μg/ml puromycin.

### Immunoblotting

Cells were harvested, and protein quantification was performed via a BCA protein assay kit (ThermoFisher). Protein samples were loaded as 20 µg/well to Mini-PROTEAN® TGX™ gels (Bio-Rad), and the transfer of proteins was performed onto the PVDF membrane. Non-specific proteins were blocked, and incubation of the PVDF membranes was carried out overnight with recommended concentrations of primary antibodies at 4 °C. Following washes, membranes were incubated with HRP-linked secondary antibodies for 1 h at room temperature. Primary and secondary antibodies are listed in Supplementary Table S3. Following final washes and incubation with ECL (ThermoFisher), the signals on blots were visualized by ChemiDoc MP Imaging System (Bio-Rad).

### Oil Red O and immunofluorescence staining

Cells were cultured on glass coverslips and fixed with 4% paraformaldehyde (PFA). Coverslips were washed with DPBS-Tween, rinsed with 60% isopropanol, and stained with Oil Red O (Sigma, USA). Cells were rinsed with 60% isopropanol and running tap water, respectively, and prepared for immunofluorescence staining steps. Permeabilization was performed in Triton X-100, and blocking of non-specific epitopes was carried out by incubation in Super Block (ScyTek, USA) medium. Thereafter, the cells were incubated with primary (overnight, 4 °C) and secondary (1 h, room temperature) antibodies. Coverslips were covered with Fluoroshield mounting medium with DAPI (Abcam, UK), and images were taken using the confocal microscope (Leica, DMI8). For triple staining, after permeabilization and blocking steps, cells and the tissue samples of the ovary and testis were incubated with LC3B (Alexa Fluor 647 Conjugate), LAMP2, and perilipin3 antibodies overnight at 4 °C. DPBS-Tween was used to wash and incubation with secondary antibodies including Alexa Fluor 568 and Alexa Fluor 488 was performed subsequently for 1 h at 37 °C. After washing with DPBS-Tween, samples were covered and visualized. All antibodies and fluorescent dyes used in this study are listed in Supplementary Table S2.

Image intensity and co-localization analyses were quantitatively performed using the ImageJ software (v2.1.0/1.53c, National Institutes of Health). The intensity and co-localization measurements using the ImageJ software were analyzed based on the following principles: Intensity measurement: intensity of selected area/vastness of selected area = mean (mean gray value) or average intensity; and co-localization measurement: colocalized particle number/nucleus number = co-localization.

### NBD-cholesterol uptake assay and Filipin staining

For NBD-cholesterol staining, cells were stained lively with 1 μg/ml NBD cholesterol along with 100 nM LysoTracker (for detection of lysosomes) for 1 h at 37 °C and 5% CO_2_. After washing, cells were fixed by 4% PFA and coated with Fluoroshield mounting medium with DAPI and visualized by confocal microscope (Leica, DMI8).

Filipin staining was performed along with LC3B primary antibody as an autophagosome marker. In the first step, cells were fixed by 4% PFA, washed with DPBS-Tween, and incubated by LC3B primary antibody (overnight at 4 °C). Then, cells were stained for 20 min with 100 μg/ml Filipin and 5 μM DRAQ5 (red nucleus dye). Afterward, cells were washed, coated with Fluoroshield mounting medium, and visualized by confocal microscope (Leica, DMI8). Image intensity and co-localization analysis methods are explained above.

### Confocal real-time live-cell imaging

For the real-time visualization of the lipid uptake and transportation process to the lysosome, the cells were live stained using 2 µM BODIPY, 100 nM LysoTracker, and 1 μg/ml Hoechst 33342. The live-cell imaging process was performed by a confocal microscope (Leica, DMI8) equipped with an incubation chamber (37 °C and 5% CO_2_).

The intensity and co-localization measurements using the ImageJ software were analyzed based on the following principles: Intensity measurement: intensity of selected area/vastness of selected area = mean (mean gray value) or average intensity; and co-localization measurement: colocalized particle number/nucleus number = co-localization.

### Cholesterol/cholesteryl ester assay

Total cholesterol isolation and free cholesterol, and cholesteryl esters quantification were performed using a colorimetric method (the Cholesterol/ Cholesteryl Ester Assay Kit, ab65359, Abcam, UK) according to the manufacturer’s instructions. Output was measured on a microplate reader (OD570nm).

### Hormone assays

Estradiol (E_2_), Progesterone (P_4_) and Testosterone (T) levels in culture media were determined by using electrochemiluminescence immunoassay “ECLIA” (Elecsys and Cobas immunoassay analyzers, Roche Diagnostics, USA). Lower detection limits and the coefficient of variation (CV, %) were 5.00 pg/ml and 1.4% for E_2_, 0.030 ng/ml and 1.2% for P_4_ and 8 ng/ml and 1.6% for T.

### Statistical analysis

Transcript levels of the steroidogenic enzymes, FSH/LH receptors, hormone levels and the signal intensities in immunoblotting and confocal imaging were continuous variables and, therefore, expressed as the mean ± SD. ANOVA/Tukey’s test or Kruskal–Wallis/Dunn post hoc tests were applied to compare parametric and non-parametric data, respectively. The sample size required for statistical significance and proper interpretation of the results was calculated based on the qRT-PCR assays and immunoblot assays. We have used the ΔΔCt method for the relative quantitation of target gene mRNAs [9, 21, 23, 24]. The significance level was set at 5% (*p* < 0.05), and GraphPad Prism version 9 was used to analyze the data and create the figures.

## Results

### Luteotropic hormones hCG and LH increase progesterone hormone production and activates autophagy in human luteinized granulosa cells and corpus luteum samples

**Fig. 1:**
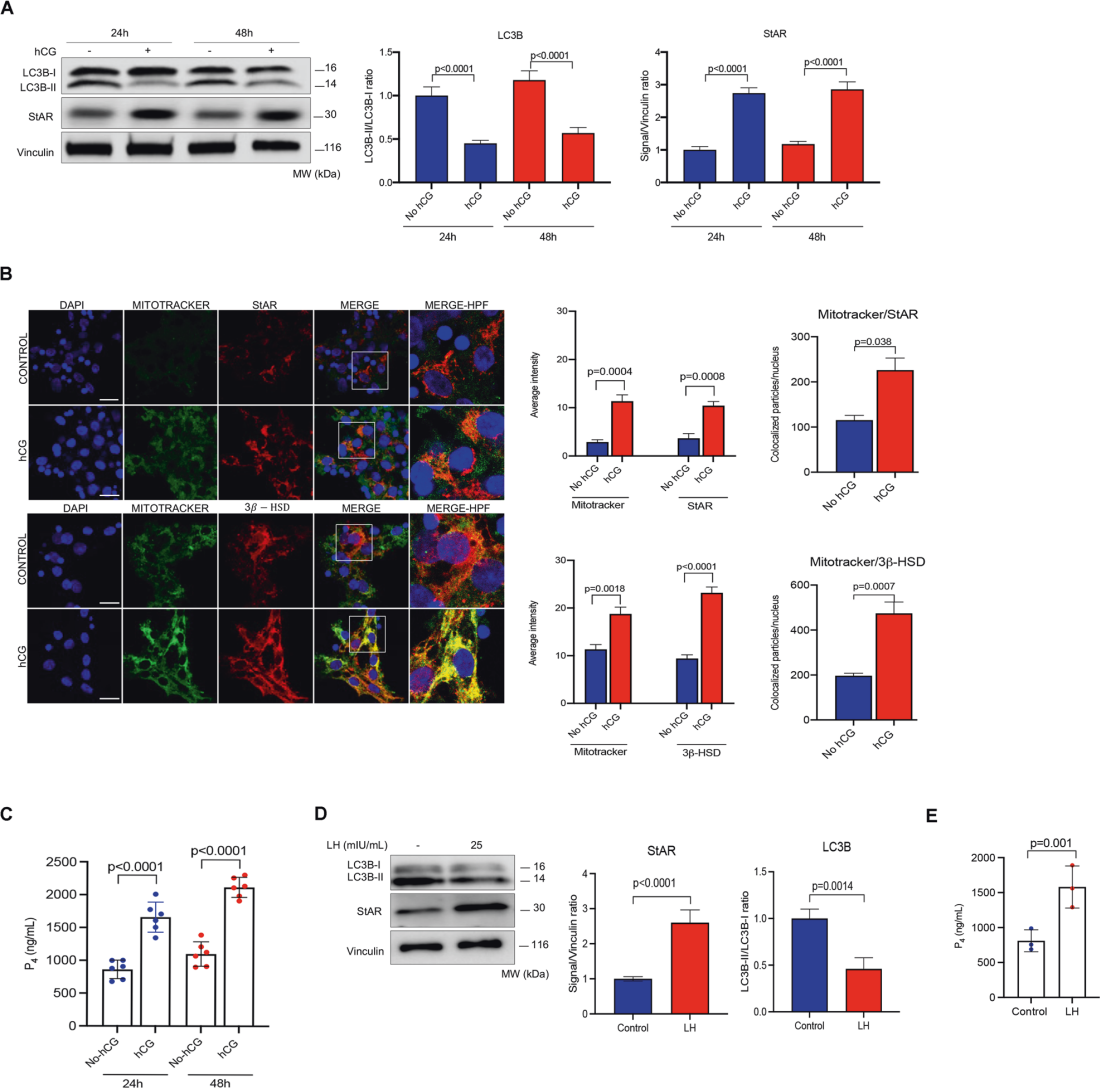
Gonadotropin hormone hCG (human chorionic gonadotropin) increases steroidogenesis and activates autophagy in granulosa cells. **A** Representative blots for indicated proteins 24 and 48 h after treatment with hCG (10 IU/ml). Densitometric quantification is indicated to the right of the blots. Mean ± SD, *N* = 6 biological replicates analyzed using one-way ANOVA, with Tukey’s test for multiple comparisons. **B** Representative confocal images of the cells after staining for the steroidogenic enzymes StAR and 3β-HSD 24 h after treatment with hCG (10 IU/ml). The insets are shown at a high power field (HPF). Quantification of the signal intensities and co-localizations of the signals are indicated to the right of the images. Nuclei stained with DAPI. Scale bars represent 20 μm. Mean ± SD, *N* = 6 biological replicates analyzed using one-way ANOVA, with Tukey’s test for multiple comparisons. **C** Representative graphic bar indicates progesterone (P_4_) hormone production of the cells before and after treatment with hCG. Mean ± SD, *N* = 6 biological replicates analyzed using one-way ANOVA, with Tukey’s test for multiple comparisons. **D** Representative blots of the luteinized granulosa cells before and 24 h after treatment with luteinizing hormone (LH) at the indicated concentration. Densitometric quantification is indicated to the right of the blots. Mean ± SD, *N* = 3 biological replicates analyzed using one-way ANOVA, with Tukey’s test for multiple comparisons. **E** In vitro progesterone (P_4_) production of the luteinized granulosa cells 24 h after treatment LH (25 mIU/ml).

Primary human luteinized GCs obtained from IVF patients can maintain their viability, steroidogenic activity and produce detectable amounts of E_2_ and P_4_ in culture for several days. They also respond to exogenously administered luteotropic hormones hCG/LH by increasing their steroidogenic activity and P_4_ output over the basal state, as previously shown by our lab and the others [9, 16, 23]. In agreement, treatment with recombinant hCG significantly upregulated the expression of StAR and 3β-HSD in the immunoblot analysis (Fig. 1A) and confocal images (Fig. 1B) and resulted in a significant increase in P_4_ production of these cells at 24 and 48 h (Fig. 1C). Interestingly, we also observed in immunoblot analysis that the expression of microtubule-associated protein 1A/1B light chain 3B-II (LC3B-II) decreased after hCG treatment (Fig. 1A). Similar results were obtained after treatment of the cells with recombinant LH (Fig. 1D, E). Given that LC3B-II itself is an autophagy substrate that degraded following the fusion of the autophagosome with lysosome, these observations suggest that hCG may induce autophagic flux and, therefore, there might be a link between autophagy and steroidogenesis in these cells.

### Pharmacological inhibition of autophagy impairs basal and hCG-stimulated progesterone production of luteinized granulosa cells and corpus luteum tissue samples

**Fig. 2:**
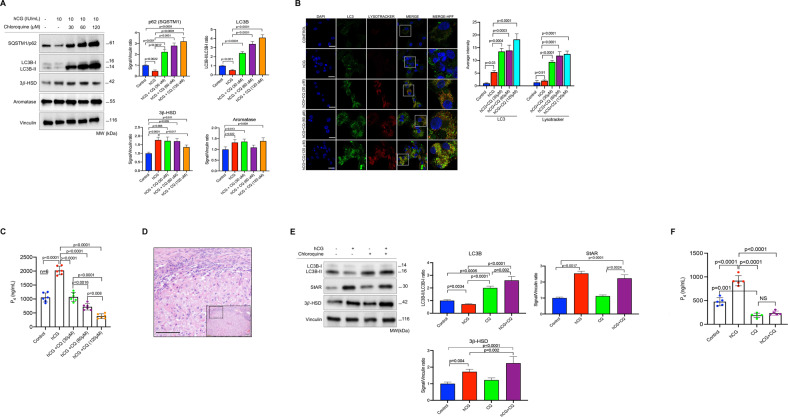
Pharmacological inhibition of autophagy with chloroquine (CQ) impairs steroidogenesis in luteinized granulosa cells and corpus luteum tissue samples. **A** Representative blots for indicated proteins after treatment of the luteinized granulosa cells with hCG w/wo chloroquine (CQ) at indicated concentrations. Densitometric quantification is indicated to the right of the blots. Mean ± SD, *N* = 6 biological replicates analyzed using one-way ANOVA, with Tukey’s test for multiple comparisons. **B** Representative confocal images of the cells stained for LC3 (green) and lysotracker (red) 24 h after treatment with hCG w/wo CQ at indicated concentrations. Quantification of the signal intensities and co-localizations of the signals are indicated to the right of the images. Nuclei stained with DAPI. Scale bars represent 20 μm. Mean ± SD, *N* = 6 biological replicates analyzed using one-way ANOVA, with Tukey’s test for multiple comparisons. **C** Representative graphic bar indicates progesterone (P_4_) production of the cells before and 24 h after treatment with hCG w/wo CQ at indicated concentrations. Mean ± SD, *N* = 6 biological replicates analyzed using one-way ANOVA, with Tukey’s test for multiple comparisons. **D** Histological section of corpus luteum (CL) tissue sample after hematoxylin-eosin staining. Scale bar represents 50 μm. **E** Representative blots for indicated proteins 24 h after treatment of the CL tissue samples with hCG w/wo CQ at indicated concentrations. Densitometric quantification is indicated to the right of the blots. Mean ± SD, *N* = 5 biological replicates analyzed using one-way ANOVA, with Tukey’s test for multiple comparisons. **F** Representative graphic bar indicates progesterone (P_4_) production of the CL tissue samples before and 24 h after treatment with hCG (10 IU/ml) w/wo CQ (60 μM). Mean ± SD, *N* = 5 biological replicates analyzed using one-way ANOVA, with Tukey’s test for multiple comparisons.

Next, the luteinized GCs were treated with a fixed dose of hCG combined with incremental concentrations of lysosomal proteolysis inhibitor chloroquine. We observed that LC3B-II and the autophagic cargo adapter protein SQSTM1/p62 accumulated in immunoblot analysis (Fig. 2A), and LC3 signal intensity and LC3/lysotracker co-localization gradually increased in the confocal images (Fig. 2B), and P_4_ production began to drop gradually (Fig. 2C) in the cells exposed to hCG+chloroquine combinations in comparison to those treated with hCG alone. This finding indicates that inhibition of autophagy with chloroquine significantly blunted hCG-induced P_4_ output. The reduction in P_4_ output was chloroquine dose-dependent, and there was an inverse correlation between the concentrations of chloroquine and the amount of P_4_ produced (*R*^2^ = 0.81, 95%CI: −0.98 to −0.60, *p* = 0.0008). Alternatively, when the cells were treated with incremental concentrations of hCG (1–5–10 IU/ml) and a fixed dose of chloroquine (60 μM), LC3B-II accumulation gradually increased in immunoblot analysis, but P_4_ production did not exhibit any meaningful increase despite hCG-induced upregulated expression of StAR (Supplementary Fig. S1A, B).

In another set of experiments, the luteinized GCs were treated only with chloroquine at the same concentrations only to see the effect of inhibition of autophagy on basal P_4_ production. We observed that the P_4_ output of the cells began to decrease gradually (Supplementary Fig. S1C) along with a gradual accumulation of LC3B-II and SQSTM1/p62 in immunoblotting (Supplementary Fig. S1D) and an increase in LC3/lysotracker co-localization in confocal imaging (Supplementary Fig. S1E). We also carried out cell death/viability and apoptosis assays to rule out the possibility that the decrease in P_4_ production is due to chloroquine-induced cell toxicity and found no difference between the control and chloroquine-treated cells in terms of Yo-Pro-1 uptake in immunofluorescence microscopy (Supplementary Fig. S1F) and cleaved PARP expression in immunoblotting (Supplementary Fig. S1D).

Treatment of the cells with another pharmacological autophagy inhibitor, vinblastine, which inhibits the fusion of the autophagosome with lysosome, yielded similar results. Basal and hCG-induced P_4_ steroidogenesis were significantly reduced in the luteinized GCs treated with vinblastine (Supplementary Fig. S1G), along with a marked accumulation of LC3B-II in immunoblot analysis (Supplementary Fig. S1H).

The effect of pharmacological inhibition of autophagy on steroidogenesis was also tested on human corpus luteum (CL) tissue samples (Fig. 2D) as an ex vivo explant culture system. Similar to what we observed in the luteinized GCs, chloroquine treatment, either alone or in combination with hCG, caused a marked accumulation of LC3B-II in immunoblotting (Fig. 2E) and resulted in a significant reduction in basal and hCG-induced P_4_ output of the samples (Fig. 2F). Taken collectively, these findings provide evidence that degradation of the autophagy-selective substrates (LC3B and SQSTM1), and therefore autophagic flux, is accelerated by luteotropic hormone hCG and that pharmacological inhibition of autophagic process impairs basal and hCG-stimulated P_4_ steroidogenesis in human luteinized granulosa cells and corpus luteum tissue samples in vitro.

We also analyzed the effects of autophagy induction on the steroidogenic function of the luteal GCs using the mTOR inhibitor drug rapamycin. The drug inhibited the mTOR pathway and accelerated autophagic flux, as evidenced by decreased expression of phospho-p70s6 and LC3B-II, respectively, in immunoblot analysis. Interestingly, basal P_4_ production was not considerably affected, but hCG-induced P_4_ output was significantly reduced in the cells after rapamycin treatment despite hCG-induced upregulated expression of the steroidogenic enzymes (Supplementary Fig. S2A, B). This phenomenon is likely to be related to the inhibition of the mTOR pathway, which plays a central role in steroidogenesis in luteal GCs, as previously shown [25].

### Silencing of the autophagy genes Beclin1 and Atg5 with siRNA and shRNA technologies reduces estrogen and progesterone production in the granulosa cells

**Fig. 3:**
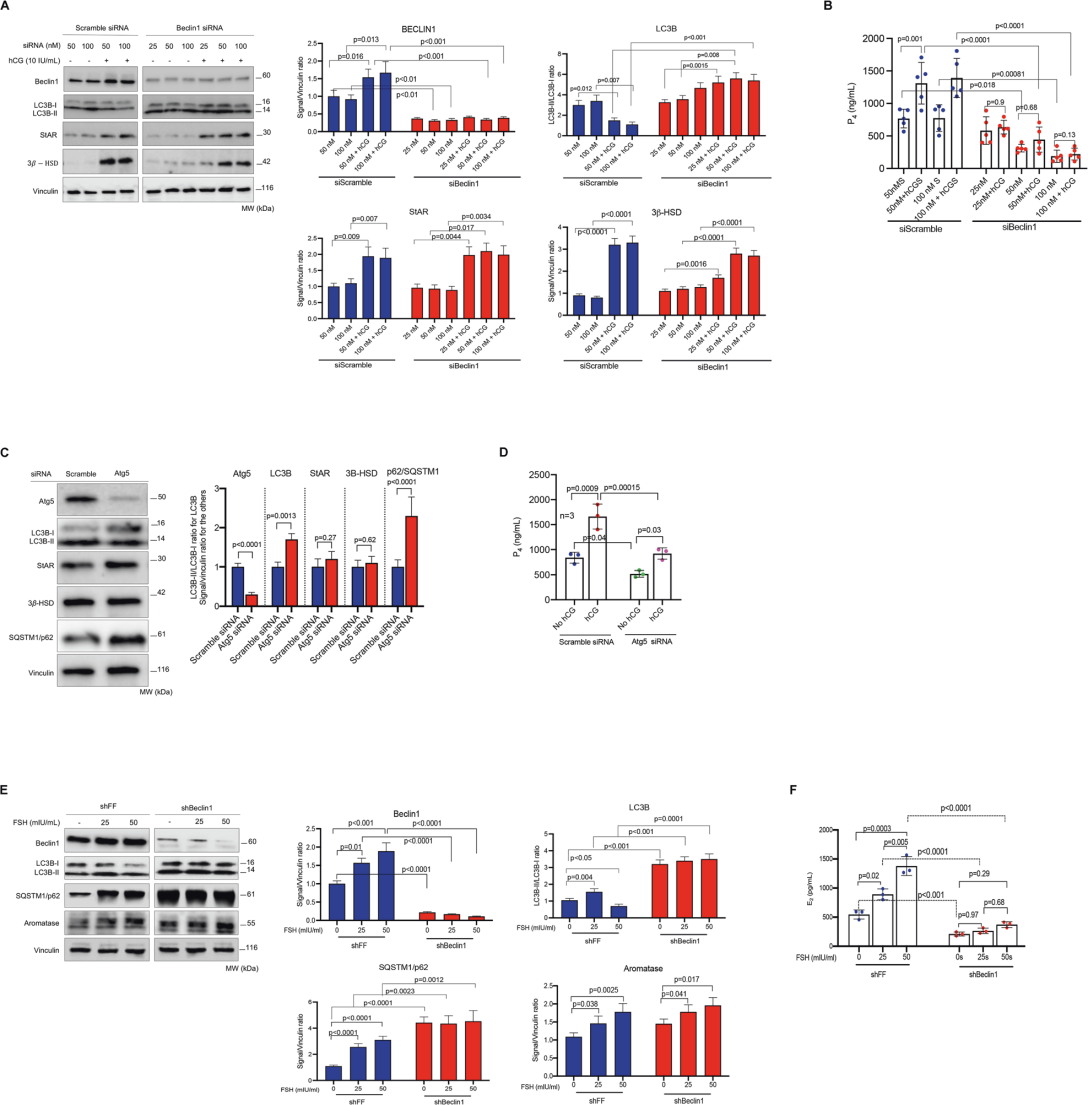
Genetic interruption of autophagy via siRNA and shRNA significantly reduces progesterone and estrogen production in granulosa cells. **A** Representative blots for indicated proteins of scramble (control) siRNA, Beclin1 siRNA and hCG-treated luteinized granulosa cells. Densitometric quantification is indicated to the right of the blots. Mean ± SD, *N* = 5 biological replicates analyzed using one-way ANOVA, with Tukey’s test for multiple comparisons. **B** Representative graphic bar indicates progesterone (P_4_) production of the luteinized granulosa cells transfected with control siRNA, Beclin1 siRNA and treated with hCG (10 IU/ml). Mean ± SD, *N* = 5 biological replicates analyzed using one-way ANOVA, with Tukey’s test for multiple comparisons. **C** Representative blots for indicated proteins of control (scramble) siRNA and Atg5 siRNA-treated luteinized granulosa cells. Densitometric quantification is indicated to the right of the blots. Mean ± SD, *N* = 3 biological replicates analyzed using one-way ANOVA, with Tukey’s test for multiple comparisons. **D** Representative graphic bars indicate progesterone (P_4_) production of the luteinized granulosa cells transfected with control siRNA, Atg5 siRNA and treated with hCG (10 IU/ml). Mean ± SD, *N* = 3 biological replicates analyzed using one-way ANOVA, with Tukey’s test for multiple comparisons. **E** Representative blots for indicated proteins of control (FF) shRNA, Beclin1 shRNA and FSH-treated non-luteinized granulosa cells (HGrC1). Densitometric quantification is indicated to the right of the blots. Mean ± SD, *N* = 3 biological replicates analyzed using one-way ANOVA, with Tukey’s test for multiple comparisons. **F** Representative graphic bars indicate estradiol (E_2_) production of the HGrC1 cells transfected with control shRNA, Beclin1 shRNA and treated with FSH at indicated concentrations. Mean ± SD, *N* = 3 replicates were analyzed using one-way ANOVA, with Tukey’s test for multiple comparisons.

Next, we analyzed the effect of genetic interruption of autophagy on the steroidogenic function of the luteinized GCs. Knocking down of the Beclin1 gene via siRNA was associated with a significant reduction in P_4_ production in these cells. Both basal and hCG-stimulated P_4_ output gradually declined with increasing concentrations of Beclin1 siRNA in comparison to those cells transfected with scramble siRNA w/wo hCG (Fig. 3A, B). While hCG significantly upregulated the expression of StAR and 3β-HSD in Beclin1 silenced cells, it failed to augment their P_4_ production. Beclin1 knockdown was also associated with a marked accumulation of LC3B-II in immunoblot analysis, and this accumulation became more evident after hCG treatment. By contrast, in the cells transfected with scramble siRNA hCG treatment was associated with decreased LC3B-II/LC3B-I ratio, upregulated Beclin1 expression together with StAR and 3β-HSD in immunoblot analysis and increased P_4_ production (Fig. 3A, B). Similar results were obtained when another autophagy gene ATG5 was knocked down with siRNA in the luteinized GCs (Fig. 3C, D).

Exogenous testosterone supplementation resulted in a marked increase in the E_2_ output of the luteinized GCs. However, we did not observe any notable reduction in E_2_ production in the luteinized GCs following either pharmacological inhibition with chloroquine and vinblastine or; after genetic interruption of autophagy (Supplementary Fig. S1I). Further, P_4_ production was not completely halted after inhibition of autophagy with either method, suggesting that an alternative autophagy-independent steroidogenic mechanism is likely to be operative in the luteinized GCs. Consistent with this, we observed that the expression of HMG-CoA, the rate-limiting enzyme in de novo cholesterol synthesis, is not affected by pharmacological inhibition (Supplementary Fig. S1J) or genetic interruption of autophagy (Supplementary Fig. S1K). Therefore, taken together, it is possible that continuing production of cholesterol and P_4_ (and possibly other 21-carbon pregnanes) might provide a sufficient amount of precursor hormones to be converted downstream into estrogen hormone, which was produced at much lower concentrations compared to progesterone (pg/ml vs. ng/ml, respectively).

In order to investigate the role of autophagy in the estrogen synthesis arm of steroidogenesis, we used mitotic non-luteinizing granulosa cells (HGrC1), which express aromatase enzyme and are capable of converting T to E_2_ when culture medium is supplemented with exogenous T. However, they do not express LH receptors and are unable to undergo luteinization and produce P_4_ [20]. Therefore it is a suitable cell line to analyze the effect of autophagy inhibition on E_2_ production.

Beclin1 knockdown with shRNA resulted in a marked accumulation of SQSTM1 and LC3B-II in immunoblot analysis and caused a significant reduction in basal and FSH-stimulated E_2_ output. Further, FSH treatment was associated with a marked reduction in LC3B-II expression in the control cells but not in the Beclin-1 silenced cells (Fig. 3E, F). Control cells transduced with FF responded to FSH normally by upregulating aromatase expression and enhancing their E_2_ production in an FSH dose-dependent manner in culture media supplemented with testosterone. By contrast, FSH-induced upregulation in aromatase expression was not accompanied by increased E_2_ production in the Beclin1 silenced cells (Fig. 3E, F).

### Autophagy regulates intracellular lipid trafficking and utilization required for steroid synthesis in granulosa cells

We observed a marked increase in the intensity of oil red O staining in the confocal images of the cells treated with chloroquine. By contrast, hCG treatment produced the opposite effect. (Supplementary Fig. S2C). Dual immunofluorescence staining revealed that oil red strongly co-localizes with lipid droplet (LD) associated protein perilipin3 (TIP47), suggesting that lipid accumulation mainly occurs in the form of LDs (Supplementary Fig. S2D). We also noticed that oil red O co-localizes with autophagy substrates LC3 and SQSTM1/p62, particularly after chloroquine treatment (Supplementary Fig. S2E, F). When intracellular cholesterol contents of the cells were measured, it appeared that inhibition of autophagy with chloroquine resulted in a significant increase in the levels of free and esterified cholesterol, while hCG produced the opposite effect (Supplementary Fig. S2G).

Taken together, these findings led us to hypothesize that autophagy may play a role in the degradation of lipids (lipophagy), which are utilized for steroid hormone synthesis in steroidogenic cells.

### Gonadotropin hormones accelerate autophagic flux and promote the association of the lipid droplets with lysosome

**Fig. 4:**
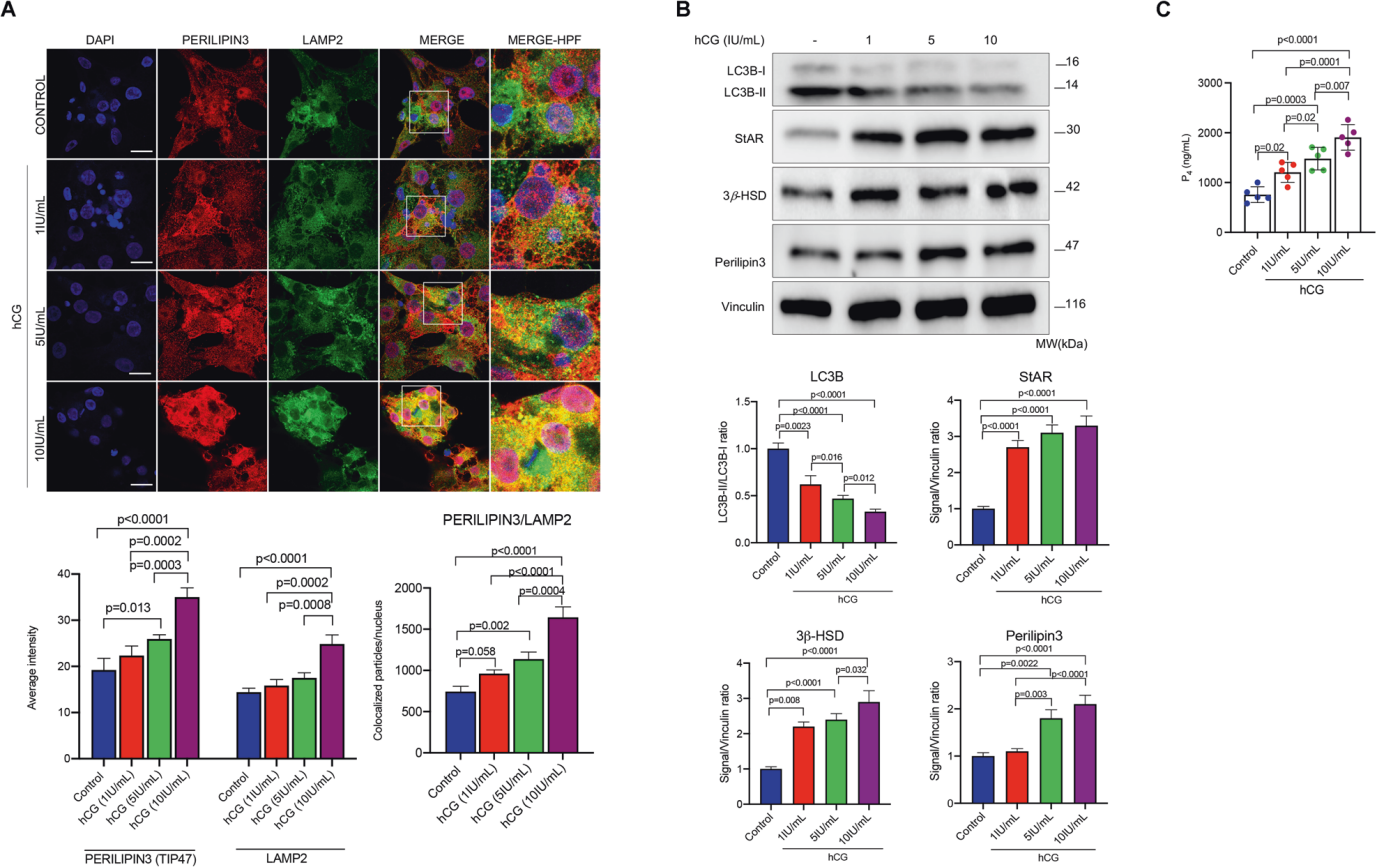
hCG promotes the association of lipid droplets with lysosomes. **A** Representative confocal images of the luteinized granulosa cells 24 h after treatment with hCG at indicated concentrations. Perilipin3 (red signal) and LAMP2 (green signal). Quantification of the signal intensities and co-localizations of the signals are indicated beneath the images. Nuclei stained with DAPI. Scale bars represent 20 μm. Mean ± SD, *N* = 5 biological replicates analyzed using one-way ANOVA, with Tukey’s test for multiple comparisons. **B** Representative blots of the luteinized granulosa cells 24 h after treatment with hCG at indicated concentrations. Densitometric quantification is indicated beneath the blots. Mean ± SD, *N* = 5 biological replicates analyzed using one-way ANOVA, with Tukey’s test for multiple comparisons. **C** Representative graphic bars indicate progesterone (P_4_) production of the luteinized granulosa cells treated with hCG at indicated concentrations. Mean ± SD, *N* = 5 biological replicates analyzed using one-way ANOVA, with Tukey’s test for multiple comparisons.

In order to test our hypothesis that autophagic degradation of intracellular lipids within the LDs occurs to provide free cholesterol for steroid hormone synthesis, we next investigated whether or not the LDs co-localize with autophagy markers/substrates and lysosomes. We observed that perilipin3/LAMP2 (lysosome-associated membrane protein-2) co-localization already exists to a certain extent in the luteinized GCs without hCG stimulation, and hCG treatment resulted in a substantial increase in this co-localization together with amplification of the signal intensity of perilipin3 in the confocal images of the cells exposed to increasing concentrations of hCG (1–5–10 IU/ml) (Fig. 4A). The expression of LC3B-II gradually diminished and perilipin3 gradually increased along with StAR and 3β-HSD expression in immunoblot analysis (Fig. 4B), and P_4_ production increased (Fig. 4C) in a dose-dependent manner after treatment with hCG at indicated concentrations. These findings suggest that the LDs associate with lysosome at basal state, and hCG augments this process.

To investigate the role of autophagy in the association of the LDs with lysosomes, we next analyzed using confocal microscopy how perilipin3/LAMP2 co-localization changes when autophagy is blocked at different stages; with chloroquine at lysosomal hydrolysis stage; with vinblastine at autophagosome-lysosome fusion stage and, with genetical interruption with Atg5 gene knockdown via siRNA via at initial stage of autophagosome formation. Chloroquine treatment resulted in a marked perilipin3 accumulation and an increase in the perilipin3/LAMP2 co-localization compared to control cells. And these effects became more evident when chloroquine was combined with hCG (Supplementary Fig. S3A). Vinblastine treatment caused perilipin3 accumulation as well. But perilipin3/LAMP2A co-localization was considerably lesser than in chloroquine-treated cells. Further, hCG addition to vinblastine did not cause such a notable increase in this co-localization (Supplementary Fig. S3A). In support of our hypothesis, we also observed in confocal analysis that perilipin3 associates with autophagosome adapter protein SQSTM1 and perilipin3/SQSTM1 co-localization increases after hCG treatment and becomes more evident when hCG is combined with either chloroquine or vinblastine (Supplementary Fig. S3B).

In order to see if perilipin3/ LAMP2A co-localization occur in a dose-dependent fashion, the cells were treated in another set of experiment with chloroquine or vinblastine at different concentrations without hCG co-treatment. We observed a gradual increase in perilipin3 signal intensity and its co-localization with LAMP2A, together with a progressive decline in P_4_ output in the cells treated with incremental concentrations of chloroquine (Supplementary Fig. S4A, B). By contrast, vinblastine administration at incremental concentrations did not cause such an increase in perilipin3/LAMP2A co-localization, although perilipin3 gradually accumulated and P_4_ output began to drop in a manner similar to chloroquine-treated cells (Supplementary Fig. S4C, D). Similarly, we did not observe any meaningful increase in perilipin3/LAMP2A co-localization even after hCG stimulation when the Atg5 gene was knocked down in the cells compared to control cells transfected with scramble siRNA, signifying the importance of autophagosome formation in the association of the LDs with the lysosome (Supplementary Fig. S4E, F). In support of our hypothesis that lipophagy regulates steroidogenesis, we monitored cholesterol trafficking in the cells and demonstrated that the uptake of NBD cholesterol, a fluorescent analog of cholesterol, is markedly enhanced after hCG treatment. Pharmacological inhibition of autophagy with chloroquine resulted in its marked cytoplasmic accumulation and a dramatic increase in its co-localization with lysotracker, particularly after hCG+chloroquine treatment (Supplementary Fig. S4G, H).

**Fig. 5:**
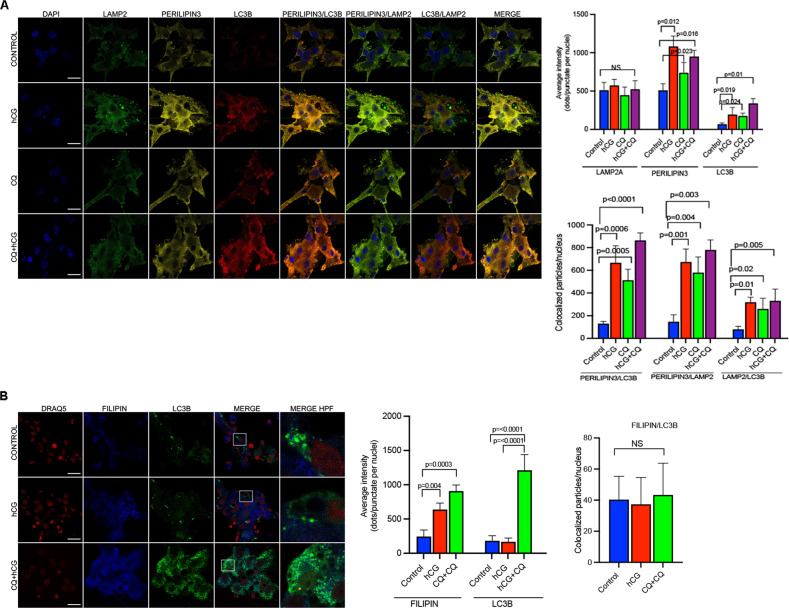
Lipid droplets but not free cholesterol associate with autophagosome. **A** Representative confocal images of the luteinized granulosa cells triple stained for LAMP2 (green signal), perilipin3 (yellow signal) and LC3 (red signal) following treatment with hCG (10 IU/ml) w/wo chloroquine (CQ, 60 μM). Quantification of the signal intensities and co-localizations of the signals are indicated to the right of the images. Nuclei stained with DAPI. Scale bars represent 20 μm. Mean ± SD, *N* = 3 biological replicates analyzed using one-way ANOVA, with Tukey’s test for multiple comparisons. **B** Representative confocal images of the luteinized granulosa cells double-stained for Filipin (blue signal) and LC3B (green signal) following treatment with hCG (10 IU/ml) w/wo chloroquine (CQ, 60 μM). Quantification of the signal intensities and co-localizations of the signals are indicated to the right of the images. Nuclei stained with DRAQ5 (red signal). Scale bars represent 20 μm. Mean ± SD, *N* = 3 biological replicates analyzed using one-way ANOVA, with Tukey’s test for multiple comparisons.

In another set of experiments, the luteinized GCs were triple stained with LC3B, perilipin3 and LAMP2 to analyze whether autophagy is involved in cholesterol trafficking. Confocal images revealed that perilipin3 co-localizes well with both LC3B and lysosome, and these co-localizations markedly increased after hCG and, particularly after treatment with hCG+ chloroquine combination (Fig. 5A, B). By contrast, the Filipin stain, which labels free cholesterol, did not co-localize with LC3B or LAMP2 in confocal images at baseline and after treatment with hCG and hCG+chloroquine combination (Fig. 5C, D). Taken collectively, these findings indicate that autophagosome seems to mediate the association of LDs with the lysosome and does not associate with free cholesterol.

In addition to hCG, we have also tested the effect of FSH on the fusion of the LDs with lysosome on both luteinized (Supplementary Fig. S5A, B) and mitotic granulosa cells (Supplementary Fig. S5C–E). In immunoblot analysis, degradation of LC3B-II gradually increased after treatment with FSH at incremental concentrations. The FSH-chloroquine combination resulted in a marked accumulation of LC3B-II. In confocal imaging, the co-localization of perilipin3 with SQSTM1 and LAMP2 increased after treatment with FSH and became more evident after treatment with an FSH+chloroquine combination.

**Fig. 6:**
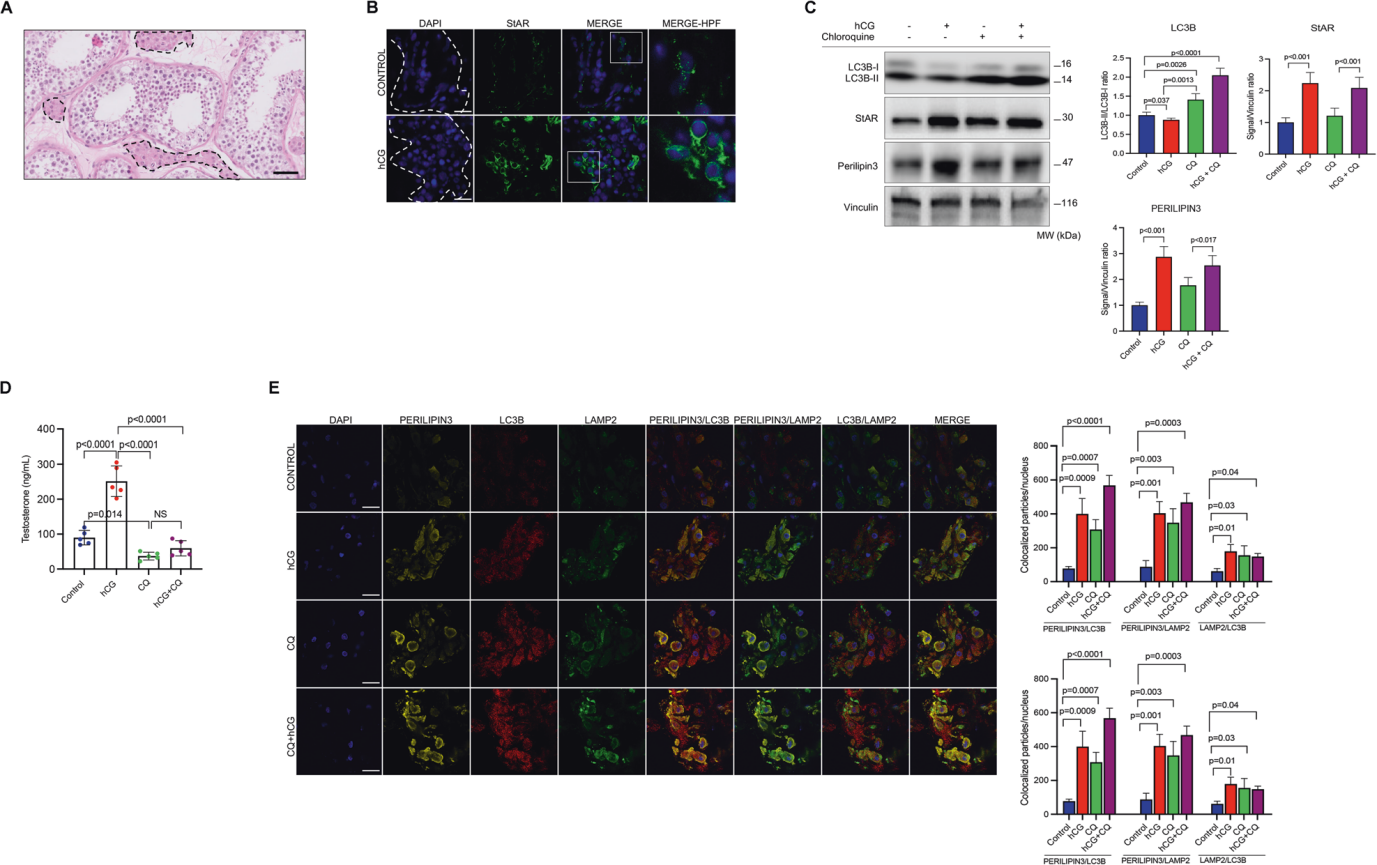
Pharmacological inhibition of autophagy with chloroquine (CQ) impairs testosterone production in testicular tissue samples. **A** Representative histological section of testicular tissue sample after hematoxylin-eosin staining. Dotted areas show the steroidogenic Leydig cells interspersed between seminiferous tubules with Sertoli cells and germ cells. Scale bar represents 50 μm. **B** Confocal images of the cryosections of the testicular tissue samples stained for StAR (green) before and after treatment with hCG (10 IU/ml). Nuclei stained with DAPI. Scale bars represent 20 μm. **C** Representative blots for indicated proteins before and after treatment with hCG (10 IU/ml) w/wo CQ (60 μM). Densitometric quantification is indicated to the right of the blots. Mean ± SD, *N* = 5 biological replicates analyzed using one-way ANOVA, with Tukey’s test for multiple comparisons. **D** Testosterone (T) production of the samples 24 h after treatment with hCG (10 IU/ml) w/wo CQ (60 μM). Mean ± SD, *N* = 5 biological replicates analyzed using one-way ANOVA, with Tukey’s test for multiple comparisons. **E** Representative confocal images of the cryosections of the testicular tissue samples 24 h after treatment with hCG (10 IU/ml) w/wo CQ (60 μM). Perilipin3 (yellow signal), LC3B (red signal) and LAMP2 (green signal). Quantification of the signal intensities and co-localizations of the signals are indicated to the right of the images. Nuclei stained with DAPI. Scale bars represent 20 μm. Mean ± SD, *N* = 5 biological replicates analyzed using one-way ANOVA, with Tukey’s test for multiple comparisons.

In order to investigate if lipophagy-mediated sex steroidogenesis is a universal process, we conducted similar experiments on the explant cultures of human testicular tissue samples (Fig. 6A). hCG treatment significantly upregulated the expression of StAR and perilipin3 in confocal imaging (Fig. 6B) and immunoblot analysis (Fig. 6C) and resulted in a drastic increase in testosterone (T) production of the samples (Fig. 6D). Inhibition of autophagy with chloroquine significantly impaired basal and hCG-induced T production along with a marked accumulation of LC3B-II in immunoblot analysis (Fig. 6C, D). Triple immunofluorescence staining of the cryosections revealed that perilipin3 already co-localizes with LC3B and LAMP2 at basal state in confocal images. hCG promotes the association of perilipin3 with LAMP2 in confocal images, and the hCG+chloroquine combination was associated with a further increase in this co-localization (Fig. 6E).

### Lipophagy-mediated steroidogenesis is compromised in patients with defective ovarian luteal function

The functions of the existing corpora luteae after ovulation are defective in a certain group of patients undergoing assisted reproduction with IVF in whom ovulation is induced with a gonadotropin-releasing hormone (GnRH) analog rather than conventionally with hCG. The luteal function is severely compromised in these patients such that the pregnancy rate significantly drops in these patients if a fresh embryo transfer is contemplated, mainly due to defective functions of the corpus luteum [10, 12, 26]. We previously demonstrated that P_4_ production is not stable and rapidly drops in the luteinized GCs of these patients, but the underlying molecular mechanism remained obscure [9]. In this regard, we hypothesized that lipophagy-mediated P_4_ steroidogenesis might be defective in these patients as a molecular explanation of this phenomenon. To this end, we carried out additional experiments to quantitatively compare the autophagic activity and steroidogenesis in the luteinized GCs obtained from normal (hCG triggered) vs. defective luteal function (GnRH analog triggered).

**Fig. 7:**
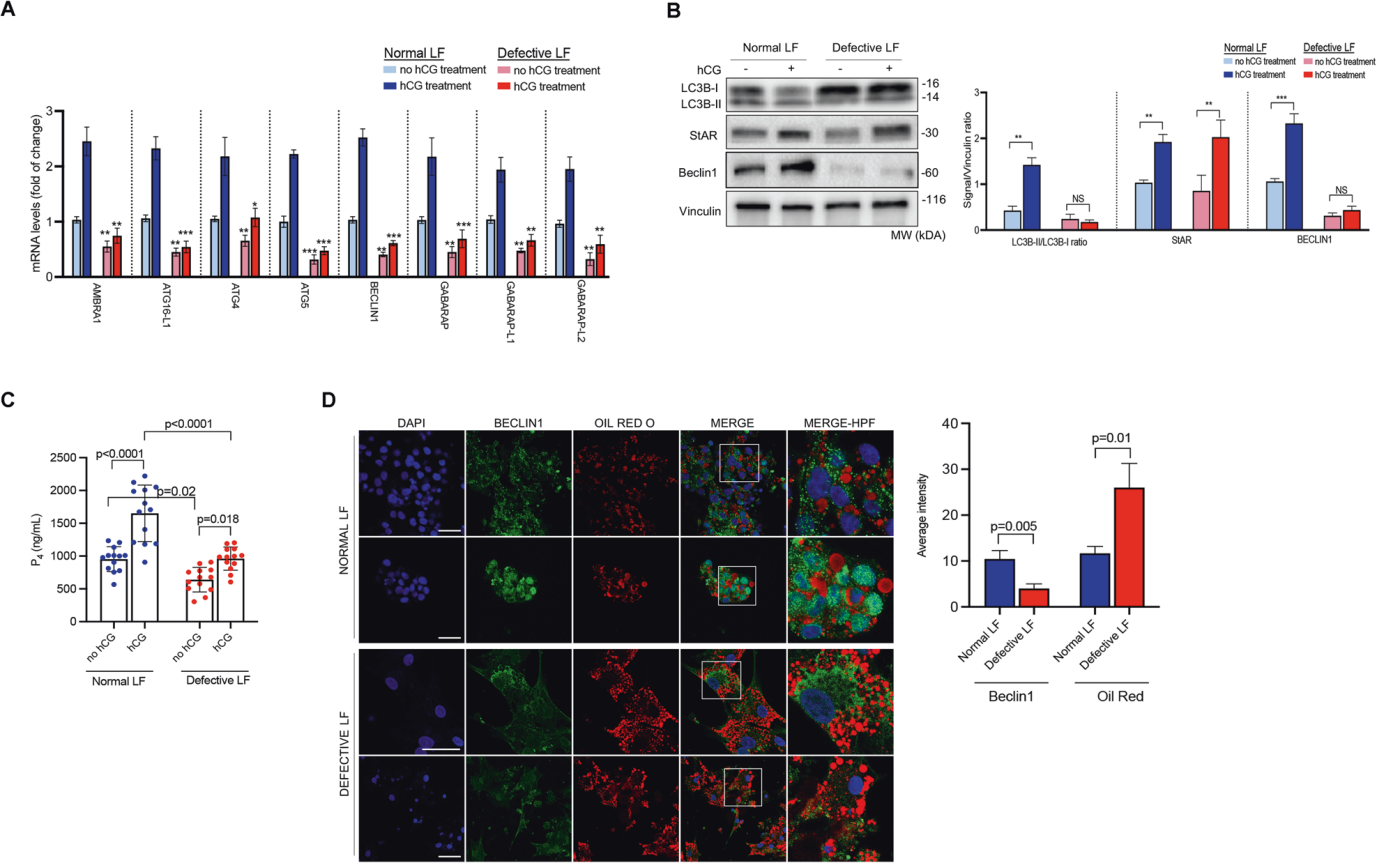
Autophagic flux is compromised in patients with defective luteal function. **A** Comparison of the transcripts of the indicated autophagy genes at baseline and 24 h after treatment with hCG (10 IU/ml) in the luteinized granulosa cells of the patients with normal and defective luteal function by quantitative RT-PCR. Mean ± SD, *N* = 13 biological replicates analyzed using one-way ANOVA, with Tukey’s test for multiple comparisons. *p < 0.01, **p < 0.001, ***p < 0.0001. **B** Representative blots for the indicated proteins at baseline and 24 h after treatment with hCG (10 IU/ml) in the luteinized granulosa cells of the patients with normal and defective luteal function. Densitometric quantification is indicated to the right of the blots. Mean ± SD, *N* = 13 biological replicates analyzed using one-way ANOVA, with Tukey’s test for multiple comparisons. NS: not significant. ***p < 0.0001. **C** In vitro progesterone (P_4_) production of the luteinized granulosa cells of the patients with normal and defective luteal function at baseline and 24 h after treatment with hCG (10 IU/ml). Mean ± SD, *N* = 13 biological replicates analyzed using one-way ANOVA, with Tukey’s test for multiple comparisons. **D** Representative confocal images of the luteinized granulosa cells stained for Beclin1 (green) and Oil Red O (red). Quantification of the signal intensities and co-localizations of the signals are indicated to the right of the images. Nuclei stained with DAPI. Scale bars represent 20 μm. Mean ± SD, *N* = 13 biological replicates analyzed using one-way ANOVA, with Tukey’s test for multiple comparisons.

**Fig. 8:**
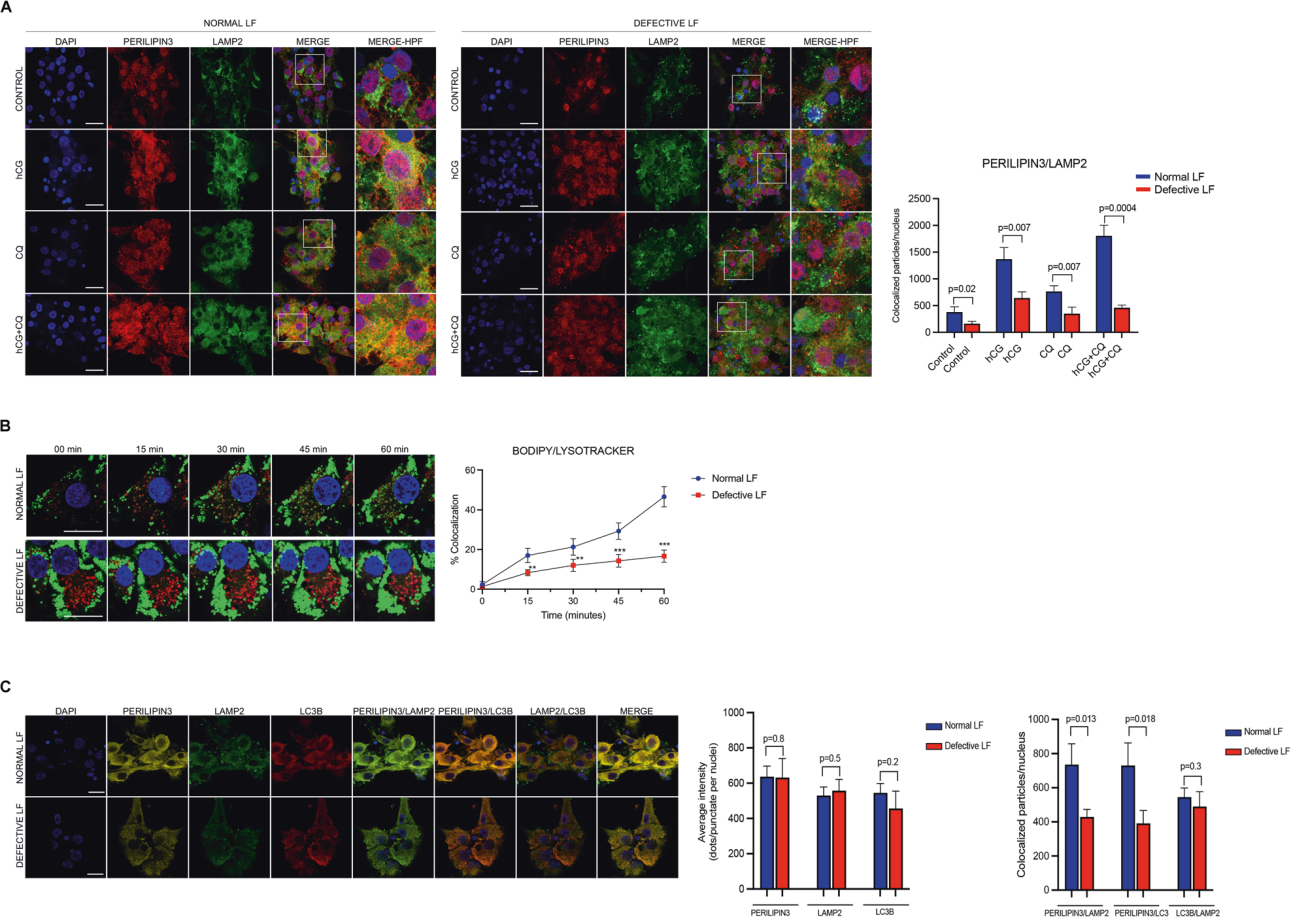
Lipophagy-mediated steroidogenesis is perturbed in patients with defective luteal function (LF). **A** Representative confocal images of the luteinized granulosa cells of the patients with normal and defective luteal function treated with hCG (10 IU/ml) w/wo chloroquine (CQ, 60 μM). Perilipin3 (red signal) and LAMP2 (green signal). Quantification of the signal intensities and co-localizations of the signals are indicated to the right of the images. Nuclei stained with DAPI. Scale bars represent 20 μm. Mean ± SD, *N* = 10 biological replicates analyzed using one-way ANOVA, with Tukey’s test for multiple comparisons. **B** Time-lapse images of confocal live microscopy of the luteinized granulosa cells of the patients with normal and defective luteal function. BODIPY (green signal) and lysotracker (red signal). Quantification and co-localizations of the signals are indicated to the right of the image. Nuclei stained with DAPI. Scale bars represent 20 μm. Mean ± SD, *N* = 10 biological replicates analyzed using one-way ANOVA, with Tukey’s test for multiple comparisons. **C** Representative confocal images of the luteinized GCs of the patients with normal and defective luteal function (LF) 24 h after treatment with hCG (10 IU/ml) w/wo CQ (60 μM). Perilipin3 (yellow signal), LC3B (red signal) and LAMP2 (green signal). Quantification of the signal intensities and co-localizations of the signals are indicated to the right of the images. Nuclei stained with DAPI. Scale bars represent 20 μm. Mean ± SD, *N* = 10 biological replicates analyzed using one-way ANOVA, with Tukey’s test for multiple comparisons.

First, we simply compared the transcripts of certain autophagy genes and found that mRNA expressions of AMBRA1, ATG16-L1, ATG4, ATG5, BECLIN1, GABARAP, GABARAP-L1/L2 were significantly downregulated in the luteinized GCs of the patients with defective luteal function. While in vitro hCG treatment resulted in a significant increase (more than two-fold) in the expression of these genes in control patients, the upregulation after hCG was less pronounced in the cells of the patients with defective luteal function (LF) (Fig. 7A). Among the genes analyzed by qRT-PCR, Beclin1 was further analyzed using confocal microscopy and immunoblot analysis. In line with qRT-PCR results, we observed that Beclin1 expression is significantly reduced in the luteinized GCs of the patients with defective LF in immunoblot analysis and did not exhibit a marked increase after hCG treatment (Fig. 7B). Furthermore, LC3B-I/B-II ratio did not meaningfully change after hCG treatment. By contrast, there was a significant reduction in the expression of LC3B-I and LC3B-II along with a concomitant increase in StAR and Beclin1 expression (Fig. 7B) and a robust increase in P_4_ output after hCG treatment in the luteinized GCs of the patients with normal LF (Fig. 7C). hCG-induced upregulation in the expression of StAR was comparable between the normal vs. defective LF groups. However, the hCG-induced increase in P_4_ production was considerably lesser in the cells with defective LF than in the control cells (Fig. 7B, C). Consistent with the qRT-PCR and immunoblot results, the signal intensity of Beclin1 was significantly weaker in the cells with defective LF in confocal image analysis (Fig. 7D). More notably, we observed in confocal images that perilipin3/LAMP2 co-localization was significantly reduced at both basal state and after hCG treatment in the cells with defective LF compared to the control cells. The defect became more evident in the cells after treatment with an hCG+chloroquine combination (Fig. 8A). We also monitored intracellular lipid trafficking in the cells using live-cell confocal time-lapse imaging technology and observed that the association of BODYPY with lysosome was significantly delayed and reduced in the cells with defective LF (Fig. 8B and Supplementary Movies S1–S4). Defective co-localization of BODYPY with lysosome became more obvious in the cells with defective LF when they were treated with an hCG+chloroquine combination (Fig. 8A, B, Supplementary Fig. S6 and Supplementary Movies S5 and S6) in comparison to the control cells with normal LF.

So far, the findings above indicate that the fusion of the LDs with lysosome is aberrantly low in the luteinized GCs of the patients with defective LF. Next, the cells were triple stained with perilipin3/LC3B/LAMP2 to see if perilipin3/LC3B co-localization is also defective in addition to perilipin3/LAMP2 co-localization in these patients. We observed that both perilipin3/LC3B and perilipin3/LAMP2 co-localizations were significantly reduced, suggesting that the observed perturbation in the fusion of the LDs with lysosome is possibly due to a defect in autophagy in these patients (Fig. 8C).

## Discussions

Several studies previously showed the link between lipophagy and sex steroidogenesis in different animal models and human testis following the first description of lipophagy in 2009 [3]. In 2014, Gavriluk et al. demonstrated, using a Beclin1 knockout mouse model, that autophagy regulates progesterone synthesis. Beclin1 knockout mouse has defective LD formation, progesterone secretion and subsequently developed preterm labor [5]. In 2019, an elegant study described autophagy-dependent steroid hormone (ecdysone) production in the *Drosophila* model by demonstrating that autophagy mediates lipid trafficking required for steroid synthesis [8]. Another well-designed study demonstrated in the mouse model that disruption of autophagy resulted in the accumulation of Na^+^/H^+^ exchanger regulatory factor2 (NHERF2) in Leydig cells, causing downregulation of scavenger receptor type 1 and defective cholesterol uptake and testosterone production. Furthermore, the investigators identified autophagy-mediated testosterone biosynthesis in human testicular samples and showed that autophagic activity was impaired in patients with low sperm count and testosterone levels [4]. Another study in 2021 demonstrated that autophagic flux and autophagy-mediated testosterone synthesis are defective in SF1-Sirt1^−^^/−^ mice. In the absence of Sirt1, LC3 fails to deacetylate and translocate to the cytoplasm, resulting in disruption of autophagic flux, impaired cholesterol uptake and deficient testosterone biosynthesis [6]. And very recently, another group of investigators demonstrated in the porcine ovary that FSH promotes progesterone production by enhancing autophagy through the upregulation of Beclin1 via the PI3K/JNK/c-Jun pathway to accelerate LD degradation in porcine GCs [7]. Another study demonstrated in a human mitotic malignant granulosa cell line (KGN) that inhibition of autophagy both genetically and pharmacologically resulted in decreased expression of genes associated with GC differentiation, including CYP19A1/Aromatase and FSHR expression and reduced estradiol synthesis. The investigators observed that when autophagy was disrupted, the transcription factor WT1 accumulated in GCs due to its insufficient degradation by the autophagic pathway, and this inhibited GC differentiation. The authors also observed that the expression of several autophagy-related genes, as well as reduced LC3-II:LC3-I and elevated SQSTM1/p62 protein levels, which are indications of decreased autophagy, were lower in GCs from biochemical premature ovarian insufficiency patients [27]. However, the investigators did not compare P_4_ production or investigate if the autophagic activity is linked to steroidogenesis in human luteinized GCs.

Consistent with the results of these papers above, we demonstrate the role of autophagy in basal and gonadotropin-stimulated sex steroid synthesis in the human ovary and testis. In brief, our findings indicate that autophagy mediates the association of the LDs with lysosomes via activating autophagosome formation to deliver the lipid cargo within the LDs to lysosomes for degradation (lipophagy) to release free cholesterol required for steroid synthesis. Lipophagy-mediated steroidogenesis appears to be operative for both basal and gonadotropin-induced production of the sex steroid hormones because pharmacological inhibition or genetic interruption of autophagy reduces both processes. Gonadotropin hormones are possibly augmenting sex steroid production by acting at several different steps of lipophagy as follows: (1) p-regulation of autophagy genes, (2) acceleration of autophagic flux, (3) increasing perilipin and LD formation and (4) promoting the association of LDs with lysosome. We summarized them in our hypothetical model of the contribution of lipophagy to sex steroid biosynthesis in the human ovary (Supplementary Fig. S7). Pharmacological inhibition or genetic interruption of autophagy did not completely halt sex steroid production in the experimental models that we tested, indicative of the presence of alternate cholesterol and/or sex steroid synthesis mechanisms in the gonads.

Defective luteal function is an important factor that substantially reduces pregnancy rates in women [10, 12, 26]. Its underlying molecular mechanisms remained obscure. We previously demonstrated that P_4_ production is not stable and rapidly drops in the luteinized GCs of these patients [9]. We have detected a number of autophagy-related abnormalities in the luteinized GCs of the patients with defective luteal function: (1) Downregulated expression of the autophagy genes, (2) incomplete degradation of LC3B-II at basal state and after gonadotropin stimulation, (3) defective cholesterol trafficking, (4) defective fusion of the LDs with lysosome and autophagosome, (5) Reduced basal and hCG-stimulated P_4_ production of these GCs analyzed. Taken together, these findings may, at least in part, provide molecular evidence for compromised P_4_ production in these patients.

Obviously, our study has several important inherent limitations. First, long-term and in-vivo effects of autophagy inhibition cannot be predicted or extrapolated from our findings because they were obtained using ex vivo and in vitro culture conditions. This is also true for the actions of gonadotropin hormones on autophagy. Although we tried to substantiate our findings using as many different tissue and cell types as possible, there are still other types of cells that produce steroid hormones. The effects of autophagy inhibition on the steroidogenic functions of those cells should be thoroughly investigated. Second, ovarian and testicular tissue samples used in the experiments were patient-derived surgical samples. They were not obtained from healthy volunteers. Therefore, the intrinsic gonadal pathologies requiring surgery might have complicated our findings even though healthy tissue parts were cautiously isolated and used for the experiments. In fact, this is a confounder and inherent universal limitation that surrounds studies that are conducted on patient-derived surgical specimens.

Sex steroid hormones control diverse physiological processes, from sexual differentiation, somatic growth, metabolic function, reproduction, and immunity to gender-specific differences in brain function and behavior [28]. Progesterone hormone is required for implantation, establishment and maintenance of pregnancy. Defective progesterone production may cause a wide range of reproductive problems, from defective implantation-related infertility to pregnancy-related complications such as miscarriage and preterm birth [29]. Estrogen hormone perhaps deserves more attention because estrogen hormone plays a critical role not only in reproduction but is also implicated in the pathogenesis of breast cancer together with progesterone hormone signaling [30] and auto-immune diseases such as systemic lupus erythematosus [31]. Similarly, androgen hormones have many reproductive and non-reproductive functions and play roles in different cardiovascular and metabolic disorders and cancers, such as prostate and liver carcinomas [32–34]. Furthermore, there are several types of gonadal tumors, such as sex cord-stromal tumors (granulosa and Leydig cell tumors), that produce high amounts of estrogen and androgen hormones and cause clinical manifestations [35]. Polycystic ovarian syndrome is one of the most common endocrinopathies of women at reproductive age, characterized by excess ovarian androgen production and androgen-related clinical manifestations such as oligomenorrhea (missed menstrual periods due to absent ovulation), hirsutism (excess hair formation in the body), obesity and insulin resistance [36]. Therefore, a detailed investigation of the role of autophagy-mediated sex steroid production in the pathogenetic mechanisms of the diseases described above is of paramount importance.

In conclusion, consistent with previous studies, our data indicate that lipophagy-mediated steroidogenesis is a general mechanism for sex steroid synthesis in the human ovary and testis and that there are some aberrations in progesterone biosynthesis by this mechanism in patients with compromised luteal function. Investigation of the role of autophagy-mediated production of sex steroid hormones might provide new insights into our understanding of the molecular mechanisms of sex hormone-related diseases and cancers to develop new alternative treatment strategies.

## Supplementary information

Supplementary data file

Supp fig-1

Supp fig-2

Supp fig-3

Supp fig-4

Supp fig-5

Supp fig-6

Supp fig-7

Movie S1

Movie S2

Movie S3

Movie S4

Movie S5

Movie S6

Original Data File

Checklist

## Data Availability

Available by request from the corresponding author or from commercial sources when applicable.

## References

[1] Miller WL (2013). Steroid hormone synthesis in mitochondria. Mol Cell Endocrinol.

[2] Wirawan E, Vanden Berghe T, Lippens S, Agostinis P, Vandenabeele P (2012). Autophagy: for better or for worse. Cell Res.

[3] Singh R, Kaushik S, Wang Y, Xiang Y, Novak I, Komatsu M (2009). Autophagy regulates lipid metabolism. Nature..

[4] Gao F, Li G, Liu C, Gao H, Wang H, Liu W (2018). Autophagy regulates testosterone synthesis by facilitating cholesterol uptake in Leydig cells. J Cell Biol.

[5] Gawriluk TR, Ko C, Hong X, Christenson LK, Rucker EB (2014). Beclin-1 deficiency in the murine ovary results in the reduction of progesterone production to promote preterm labor. Proc Natl Acad Sci USA.

[6] Khawar MB, Liu C, Gao F, Gao H, Liu W, Han T (2021). Sirt1 regulates testosterone biosynthesis in Leydig cells via modulating autophagy. Protein Cell.

[7] Liu Q, Gao H, Yang F, Zhang H, Zeng S (2021). FSH promotes progesterone synthesis by enhancing autophagy to accelerate lipid droplet degradation in porcine granulosa cells. Front Cell Dev Biol.

[8] Texada MJ, Malita A, Christensen CF, Dall KB, Faergeman NJ, Nagy S (2019). Autophagy-mediated cholesterol trafficking controls steroid production. Dev Cell.

[9] Bildik G, Akin N, Seyhan A, Esmaeilian Y, Yakin K, Keles I (2019). Luteal granulosa cells from natural cycles are more capable of maintaining their viability, steroidogenic activity and LH receptor expression than those of stimulated IVF cycles. Hum Reprod.

[10] Yding Andersen C, Vilbour, Andersen K (2014). Improving the luteal phase after ovarian stimulation: reviewing new options. Reprod Biomed Online.

[11] Shapiro BS, Andersen CY (2015). Major drawbacks and additional benefits of agonist trigger—not ovarian hyperstimulation syndrome related. Fertil Steril.

[12] Kolibianakis EM, Schultze-Mosgau A, Schroer A, van Steirteghem A, Devroey P, Diedrich K (2005). A lower ongoing pregnancy rate can be expected when GnRH agonist is used for triggering final oocyte maturation instead of HCG in patients undergoing IVF with GnRH antagonists. Hum Reprod.

[13] Bildik G, Akin N, Esmaeilian Y, Hela F, Yildiz CS, Iltumur E (2020). Terminal differentiation of human granulosa cells as luteinization is reversed by activin-A through silencing of JNK pathway. Cell Death Discov.

[14] Bildik G, Akin N, Senbabaoglu F, Sahin GN, Karahuseyinoglu S, Ince U (2015). GnRH agonist leuprolide acetate does not confer any protection against ovarian damage induced by chemotherapy and radiation in vitro. Hum Reprod.

[15] Bildik G, Esmaeilian Y, Hela F, Akin N, Iltumur E, Yusufoglu S (2022). Cholesterol uptake or trafficking, steroid biosynthesis, and gonadotropin responsiveness are defective in young poor responders. Fertil Steril.

[16] Bagnjuk K, Mayerhofer A (2019). Human luteinized granulosa cells—a cellular model for the human corpus luteum. Front Endocrinol.

[17] Li SJ, Chang HM, Wang JH, Yang J, Leung PCK (2022). The interleukin-6 trans-signaling promotes progesterone production in human granulosa-lutein cells. Biol Reprod.

[18] Henderson KM, McNatty KP (1977). A possible interrelationship between gonadotrophin stimulation and prostaglandin F2alpha inhibition of steroidogenesis by granulosa-luteal cells in vitro. J Endocrinol.

[19] Kohen P, Castro O, Palomino A, Munoz A, Christenson LK, Sierralta W (2003). The steroidogenic response and corpus luteum expression of the steroidogenic acute regulatory protein after human chorionic gonadotropin administration at different times in the human luteal phase. J Clin Endocrinol Metab.

[20] Bayasula, Iwase A, Kiyono T, Takikawa S, Goto M, Nakamura T (2012). Establishment of a human nonluteinized granulosa cell line that transitions from the gonadotropin-independent to the gonadotropin-dependent status. Endocrinology.

[21] Oktem O, Akin N, Bildik G, Yakin K, Alper E, Balaban B (2017). FSH stimulation promotes progesterone synthesis and output from human granulosa cells without luteinization. Hum Reprod.

[22] Oktem O, Bildik G, Senbabaoglu F, Lack NA, Akin N, Yakar F (2016). Cytotoxicity and mitogenicity assays with real-time and label-free monitoring of human granulosa cells with an impedance-based signal processing technology intergrating micro-electronics and cell biology. Reprod Toxicol.

[23] Bildik G, Akin N, Esmaeilian Y, Hela F, Yakin K, Onder T (2020). hCG improves luteal function and promotes progesterone output through the activation of JNK pathway in the luteal granulosa cells of the stimulated IVF cycles. Biol Reprod.

[24] Livak KJ, Schmittgen TD (2001). Analysis of relative gene expression data using real-time quantitative PCR and the 2^−ΔΔCT^ method. Methods..

[25] Moravek MB, Shang M, Menon B, Menon K (2016). HCG-mediated activation of mTORC1 signaling plays a crucial role in steroidogenesis in human granulosa lutein cells. Endocrine..

[26] Humaidan P, Quartarolo J, Papanikolaou EG (2010). Preventing ovarian hyperstimulation syndrome: guidance for the clinician. Fertil Steril.

[27] Shao T, Ke H, Liu R, Xu L, Han S, Zhang X (2022). Autophagy regulates differentiation of ovarian granulosa cells through degradation of WT1. Autophagy..

[28] Miller WL, Auchus RJ (2011). The molecular biology, biochemistry, and physiology of human steroidogenesis and its disorders. Endocr Rev.

[29] Taraborrelli S (2015). Physiology, production and action of progesterone. Acta Obstet Gynecol Scand.

[30] Scabia V, Ayyanan A, De Martino F, Agnoletto A, Battista L, Laszlo C (2022). Estrogen receptor positive breast cancers have patient specific hormone sensitivities and rely on progesterone receptor. Nat Commun.

[31] Oktem O, Yagmur H, Bengisu H, Urman B (2016). Reproductive aspects of systemic lupus erythematosus. J Reprod Immunol.

[32] Kelly DM, Jones TH (2013). Testosterone: a metabolic hormone in health and disease. J Endocrinol.

[33] Green SM, Mostaghel EA, Nelson PS (2012). Androgen action and metabolism in prostate cancer. Mol Cell Endocrinol.

[34] Kanda T, Yokosuka O (2015). The androgen receptor as an emerging target in hepatocellular carcinoma. J Hepatocell Carcinoma.

[35] Schultz KA, Schneider DT, Pashankar F, Ross J, Frazier L (2012). Management of ovarian and testicular sex cord-stromal tumors in children and adolescents. J Pediatr Hematol Oncol.

[36] Legro RS, Arslanian SA, Ehrmann DA, Hoeger KM, Murad MH, Pasquali R (2013). Diagnosis and treatment of polycystic ovary syndrome: an Endocrine Society clinical practice guideline. J Clin Endocrinol Metab.

