# Supplementary material for: Autophagy regulates sex steroid hormone synthesis through lysosomal degradation of lipid droplets in human ovary and testis
Source: Cell Death Dis. 2023 May 26;14(5):342. doi: 10.1038/s41419-023-05864-3 (PMC10220221; doi:10.1038/s41419-023-05864-3)
Supplement: Supplementary file 1 — Supplementary data file [file 41419_2023_5864_MOESM1_ESM.docx]

**SUPPLEMENTARY DATA**

Table S1: Demographic characteristics of the donor patients whose cells or gonadal tissue samples were used for the experiments

| LUTEINIZED GRANULOSA CELLS-IVF PATIENTS | | | | |
| --- | --- | --- | --- | --- |
|  | CONTROL GnRH antagonist protocol (hCG trigger) | GnRH antagonist protocol (GnRH analog trigger) | | P |
| N | 48 | 47 | |  |
| Age | 31.7±5.2 | 32.6±4.8 | | 0.15 |
| Starting gonadotropin dose | 295.3±52 | 240.3±72 | | 0.32 |
| Duration of stimulation | 10.2±1.2 | 11.3±2.1 | | 0.17 |
| Peak serum E_2_ level (ovulation trigger day) | 2808±507 | 3876±743 | | 0.002 |
| Peak Serum P_4_ level (ovulation trigger day) | 0.70±0.2 | 1.4±0.7 | | 0.006 |
| Total number of oocytes collected | 11.7±3.8 | 18.2±4.5 | | 0.005 |
| CORPUS LUTEUM TISSUE SAMPLE DONORS | | | | |
| Indications for surgery | Ovarian cryopreservation  (n=3) | Ovarian  Dermoid cysts (n=1) | Ovarian endometriomas  (n=1) | P |
| Age | 34.6±2.2 | 35.1±1.2 | 30.2±3.6 | 0.26 |
| Menstrual cycle day at the time of operation | 17.2±1.7 | 15 | 16 | 0.34 |
| TESTICULAR TISSUE SAMPLE DONORS | | | | |
| Indications for surgery (orchiectomy) | Testicular cancers  Seminomas (n=4). Yolk sac tumor (n=1) | | | P |
| Age | 22.4±5.5 | 21 | | 0.54 |
|  |  |  | |  |

**Table S2:** Primers used in the qRT-PCR assay

| **Gene** |  | **3’-Sequence-5’** |
| --- | --- | --- |
| GAPDH | Forward | ATGGAAATCCCATCACCATCTT |
|  | Reverse | CGCCCCACTTGATTTTGG |
| Ambra1 | Forward | TCAGGAGGAGGCTGCTGGAAT |
|  | Reverse | TGGAGCGCTGGCGAATACTG |
| Beclin1 | Forward | TCCCGAGGTGAAGAGCATCG |
|  | Reverse | TCGCCTGGGCTGTGGTAAGT |
| Atg4 | Forward | TGGATCCTTCAGTTGCATTGGG |
|  | Reverse | CAATGAATTCTGCCCCAGTGGT |
| Atg5 | Forward | AAGCAACTCTGGATGGGATTGC |
|  | Reverse | TGCAGCCACAGGACGAAACA |
| GABARAP | Forward | CCGAGCTGAGGATGCCTTGT |
|  | Reverse | GCAGCTTCACAGACCGTAGACA |
| GABARAP-L1 | Forward | AACAACACCATCCCTCCCACCA |
|  | Reverse | TCCCATCTGCTGGGCTTCCAA |
| GABARAP-L2 | Forward | AGGCGATCTTCCTGTTTGTGG |
|  | Reverse | GTTACGGTGCACCTAGCCCA |

**Table S3:** Antibodies and fluorescent dyes used in the study.

| **Antibody** | **Manufacturer** | **Cat. No.** |
| --- | --- | --- |
| SQSTM1/P62 | Abcam | ab56416 |
| Atg5/APG5L | Cell Signaling | 12994 |
| Beclin1 | Abcam | ab207612 |
| Perilipin3 | Abcam | ab47638 |
| Lamp2 | Abcam | ab25631 |
| StAR | Santa Cruz Biotechnology | sc166821 |
| 3β-HSD | Santa Cruz Biotechnology | sc100466 |
| Vinculin | Santa Cruz Biotechnology | sc25336 |
| HMGCR | Abcam | ab242315 |
| p70 S6 Kinase | Cell Signaling | 9202 |
| Phospho-p70 S6 Kinase | Cell Signaling | 97596 |
| LC3A/B | Cell Signaling | 12741 |
| LC3B-Alexa Fluor 647 Conjugate | Cell Signaling | 18577 |
| LC3B | Cell Signaling | 83506 |
| Cleaved PARP | Cell Signaling | 5625 |
| Aromatase (CYP19A) | Santa Cruz Biotechnology | sc-374176 |
| Mitotracker | Invitrogen | M7514 |
| Lysotracker | Invitrogen | L7528 |
| NBD-Cholesterol | Invitrogen | N1148 |
| Filipin | Sigma | F9765 |
| BODIPY | Invitrogen | D3922 |
| Hoechst 33342 | Cell Signaling | 4082 |
| DRAQ5 | BioLegend | 424101 |
| Goat anti-Moues Alexa Flour 594 secondary antibody | ThermoFisher | A11005 |
| Goat anti-Rabbit Alexa Flour 594 secondary antibody | ThermoFisher | A11012 |
| Goat anti-Mouse Alexa Flour 488 secondary antibody | ThermoFisher | A11001 |
| Goat anti-Rabbit Alexa Flour 488 secondary antibody | ThermoFisher | A11008 |
| Goat anti-Rabbit Alexa Flour 568 secondary antibody | ThermoFisher | A11011 |
| Anti-Mouse IgG, HRP-linked antibody | Cell Signaling | 7076 |
| Anti-Rabbit IgG, HRP-linked antibody | Cell Signaling | 7074 |

**Production of plasmids and viral supernatants**

Beclin1 cDNA was amplified from dsRed-Beclin1 plasmid (Addgene) with following primer set: EcoRI-adding-forward-primer (5’-CAAGCTTCGAATTCTATGGAAG-3’) and XbaI-adding-reverse-primer (5’-AGTAGATCTCCGGTGGATCCTTATTT-3’). Beclin1 coding sequence was cloned into pENTR1A no ccDB (Addgene) via EcoRI-XbaI cut sites and transferred into lentiviral expression plasmid pLEX-307 (Addgene) via LR cloning (Invitrogen). DsRed-Beclin1 cDNA was amplified from dsRed-Beclin1 plasmid (Addgene) with following primer set: KpnI-adding-forward-primer (5’-TAGGTACCATGGCCTCCTCCGA-3’) and XbaI-adding-reverse-primer (5’-AGTAGATCTCCGGTGGATCCTTATTT-3’). DsRed-Beclin1 fusion protein coding sequence was cloned into pENTR1A no ccDB (Addgene) via KpnI-XbaI cut sites and transferred into lentiviral expression plasmid pLEX-307 (Addgene) via LR cloning (Invitrogen). All vectors were confirmed by Sanger sequencing using pENTR1A-forward-sequencing-primer (5’-CTACAAACTCTTCCTGTTAGTTAG-3’) and pENTR1A-reverse-sequencing-primer (5’-ATGGCTCATAACACCCCTTG-3’). eGFP expressing pLEX-307_GFP was used as a control plasmid. shRNAs were designed and cloned into the MSCV-PM retroviral vector as previously described [5]. Firefly Luciferase-targeting shRNA (FF shRNA) was used as a control plasmid. All vectors were confirmed by Sanger sequencing using MSCV-forward-sequencing-primer (5’-CCCTTGAACCTCCTCGTTCGACCT-3’). Beclin1 shRNA sequence: TGCTGTTGACAGTGAGCGACAGGAGGAAGAGACTAACTCATAGTGAAGCCACAGATGTATGAGTTAGTCTCTTCCTCCTGGTGCCTACTGCCTCGGA.

**Production of Viral Supernatants**

HEK-293T cells were plated and transfected with 2.5 µg/ml viral vector and 0.25 µg pCMV-VSV-G (Addgene) using 20 µl FUGENE 6 (Promega). Supernatants were collected 48 hr and 72 hr post-transfection and filtered through 0.45-µm pore size filters. To concentrate the viruses, viral supernatants were mixed with PEG8000 (Sigma, dissolved in DPBS, 10% final concentration) and left overnight at 4 °C. The next day, supernatants were centrifuged at 2500 rpm for 20 min, and pellets were re-suspended in PBS for viral transduction.

**SUPPLEMENTARY FIGURE LEGENDS**

**Fig. S1:** (A) Representative blots of the luteinized granulosa cells before and 24 hours after treatment with hCG with chloroquine (CQ) at indicated concentration. Densitometric quantification is indicated to the right of the blots. Mean ± SD, N = 4 biological replicates analyzed using one-way ANOVA, with Tukey’s test for multiple comparisons. (B) In vitro progesterone (P_4_) production of the luteinized granulosa cells 24 hours after treatment LH (25 mIU/mL). (C) In vitro progesterone (P_4_) production of the luteinized granulosa cells 24 hours after treatment with CQ at indicated concentrations. Mean ± SD, N = 4 biological replicates analyzed using one-way ANOVA, with Tukey’s test for multiple comparisons. (D) Representative blots for indicated proteins before and after treatment of the luteinized granulosa cells with CQ at indicated concentrations. Densitometric quantification is indicated to the right of the blots. Mean ± SD, N = 3 biological replicates analyzed using one-way ANOVA, with Tukey’s test for multiple comparisons. (E) Representative confocal images of the luteinized granulosa cells treated with CQ at indicated concentrations. LC3 (green signal) and Lysotracker (red signal). Quantification and co-localizations of the signals are indicated to the right of the image. Nuclei stained with DAPI. Scale bars represent 20 μm. Mean ± SD, N = 3 biological replicates analyzed using one-way ANOVA, with Tukey’s test for multiple comparisons. (F) Viability assay with intra-vital Yo-PRO-1 staining (green signal) in the luteinized granulosa cells treated with CQ at indicated concentrations. LC3 (green signal) and Lysotracker (red signal). Nuclei stained with Hoechst 33342. Yo-pro-1 positive cells (%) expressed after a minimum 100 cells at four different areas of low power field. (G) In vitro progesterone (P_4_) production of the luteinized granulosa cells 24 hours after treatment with hCG (10 IU/mL) w/wo vinblastine (20 μM). Mean ± SD, N = 3 biological replicates analyzed using one-way ANOVA, with Tukey’s test for multiple comparisons. (H) Representative blots for the indicated proteins of the luteinized granulosa cells treated with hCG (10 IU/mL) w/wo vinblastine (20 μM). Densitometric quantification is indicated to the right of the blots. Mean ± SD, N = 3 biological replicates analyzed using one-way ANOVA, with Tukey’s test for multiple comparisons. (I) Representative graphic bars indicate estradiol (E_2_) production of the luteinized granulosa cells treated with indicated drugs w/wo testosterone (T) supplementation. Mean ± SD, N = 3 biological replicates analyzed using one-way ANOVA, with Tukey’s test for multiple comparisons. (J) Representative blot for indicated proteins of the luteinized granulosa cells treated with hCG (10 IU/mL) w/wo chloroquine (60 μM). (K) Representative blot for indicated proteins of the control (scramble) siRNA and Atg5 siRNA treated luteinized granulosa cells.

**Fig. S2:** (A) Representative blots for indicated proteins of the luteinized granulosa cells treated with hCG w/wo rapamycin at indicated concentrations. Densitometric quantification is indicated to the right of the blots. Mean ± SD, N = 4 biological replicates analyzed using one-way ANOVA, with Tukey’s test for multiple comparisons. (B) Representative graphic bars indicate progesterone (P_4_) production of the luteinized granulosa cells treated with hCG w/wo rapamycin. Mean ± SD, N = 4 biological replicates analyzed using one-way ANOVA, with Tukey’s test for multiple comparisons. (C) Confocal images of the luteinized granulosa cells treated with hCG (10 IU/mL) w/wo CQ (60 μM). Mitotracker (green signal) and Oil Red O (red signal). Quantification and co-localizations of the signals are indicated beneath the image. Nuclei are stained with DAPI. Scale bars represent 20 µm. Mean ± SD, N = 3 biological replicates analyzed by one-way ANOVA, with Tukey’s test for multiple comparisons. (D) Confocal images of the luteinized granulosa cells treated with hCG (10 IU/mL) w/wo CQ (60 μM). Perilipin3 (green signal) and Oil Red O (red signal). Quantification and co-localizations of the signals are indicated beneath the image. Nuclei are stained with DAPI. Scale bars represent 20 µm. Mean ± SD, N = 3 biological replicates analyzed by one-way ANOVA, with Tukey’s test for multiple comparisons. (E) Confocal images of the luteinized granulosa cells treated with hCG (10 IU/mL) w/wo CQ (60 μM). LC3 (green signal) and Oil Red O (red signal). Quantification and co-localizations of the signals are indicated beneath the image. Nuclei are stained with DAPI. Scale bars represent 20 µm. Mean ± SD, N = 3 biological replicates analyzed by one-way ANOVA, with Tukey’s test for multiple comparisons. (F) Confocal images of the luteinized granulosa cells treated with hCG (10 IU/mL) w/wo CQ (60 μM). SQSTM1/p62 (green signal) and Oil Red O (red signal). Quantification and co-localizations of the signals are indicated beneath the image as graphic bars. Nuclei are stained with DAPI. Scale bars represent 20 µm. Mean ± SD, N = 3 biological replicates analyzed by one-way ANOVA, with Tukey’s test for multiple comparisons. (G). Representative graphic bars indicate total, free and esterified cholesterol levels in the luteinized granulosa cells before and 24 hours after treatment with hCG (10 IU/mL) w/wo CQ (60 μM). Mean ± SD, N = 3 biological replicates analyzed by one-way ANOVA, with Tukey’s test for multiple comparisons.

**Fig. S3:** (A) Representative confocal images of the luteinized granulosa cells treated with hCG (10 IU/mL) w/wo CQ (60 μM) and vinblastine (20 μM). Perilipin3 (red signal) and LAMP2 (green signal). Quantification and co-localizations of the signals are indicated beneath the image. Nuclei are stained with DAPI. Scale bars represent 20 µm. Mean ± SD, N = 5 biological replicates analyzed by one-way ANOVA, with Tukey’s test for multiple comparisons. (B) Confocal images of the luteinized granulosa cells treated with hCG (10 IU/mL) w/wo CQ (60 μM) and vinblastine (20 μM). Perilipin3 (red signal) and SQSTM1 (green signal). Quantification and co-localizations of the signals are indicated beneath the image. Nuclei are stained with DAPI. Scale bars represent 20 µm. Mean ± SD, N = 4 biological replicates analyzed by one-way ANOVA, with Tukey’s test for multiple comparisons.

**Fig. S4:** (A) Representative confocal images of the luteinized granulosa cells treated with chloroquine (CQ) at indicated concentrations. Perilipin3 (red signal) and LAMP2 (green signal). Co-localizations of the signals are indicated as graphic bars beneath the image. Nuclei are stained with DAPI. Scale bars represent 20 µm. Mean ± SD, N = 3 biological replicates analyzed by one-way ANOVA, with Tukey’s test for multiple comparisons. (B) In-vitro progesterone (P_4_) production of the cells treated with CQ at indicated concentrations. Mean ± SD, N = 3 biological replicates analyzed using one-way ANOVA, with Tukey’s test for multiple comparisons. (C) Representative confocal images of the luteinized granulosa cells treated with vinblastine at indicated concentrations. Perilipin3 (red signal) and LAMP2 (green signal). Co-localizations of the signals are indicated beneath the image. Nuclei are stained with DAPI. Scale bars represent 20 µm. Mean ± SD, N = 3 biological replicates analyzed by one-way ANOVA, with Tukey’s test for multiple comparisons. (D) In-vitro progesterone (P_4_) production of the cells treated with vinblastine at indicated concentrations. Mean ± SD, N = 3 biological replicates analyzed using one-way ANOVA, with Tukey’s test for multiple comparisons. (E) Representative confocal images of the luteinized granulosa cells treated with control (scramble) siRNA, Atg5 siRNA w/wo hCG (10 IU/mL). Perilipin3 (red signal) and LAMP2 (green signal). Co-localizations of the signals are indicated beneath the image. Nuclei are stained with DAPI. Scale bars represent 25 µm. Mean ± SD, N = 3 biological replicates analyzed by one-way ANOVA, with Tukey’s test for multiple comparisons. (F) In-vitro progesterone (P_4_) production of the cells treated with control (scramble) siRNA, Atg5 siRNA w/wo hCG. Mean ± SD, N = 3 biological replicates analyzed using one-way ANOVA, with Tukey’s test for multiple comparisons. (G) Representative confocal images of the luteinized granulosa cells treated with hCG (10 IU/mL) w/wo CQ (60 μM). Lysotracker (red signal) and NBD cholesterol (green signal). Quantification and co-localizations of the signals are indicated beneath the image. Nuclei are stained with DAPI. Scale bars represent 20 µm. Mean ± SD, N = 4 biological replicates analyzed by one-way ANOVA, with Tukey’s test for multiple comparisons. (H) In-vitro progesterone (P_4_) production of the cells treated with hCG (10 IU/mL) w/wo CQ (60 μM). Mean ± SD, N = 3 biological replicates analyzed by one-way ANOVA, with Tukey’s test for multiple comparisons.

**Fig. S5:** (A) Representative blots for indicated proteins of the luteinized granulosa cells treated with FSH w/wo chloroquine (CQ) at indicated concentrations. Densitometric quantification is indicated beneath the blot. Mean ± SD, N = 3 biological replicates analyzed using one-way ANOVA, with Tukey’s test for multiple comparisons. (B) Representative confocal images of the luteinized granulosa cells treated with FSH w/wo CQ. Perilipin3 (red signal) and LAMP2 (green signal). Co-localizations of the signals are indicated to the right of the image. Nuclei are stained with DAPI. Scale bars represent 20 µm. Mean ± SD, N = 3 biological replicates analyzed by one-way ANOVA, with Tukey’s test for multiple comparisons. (C) Representative blots for indicated proteins of the mitotic (HGrC1) granulosa cells treated with FSH w/wo CQ at indicated concentrations. Densitometric quantification is indicated beneath the blot. Mean ± SD, N = 3 biological replicates analyzed using one-way ANOVA, with Tukey’s test for multiple comparisons. (D) Representative confocal images of the HGrC1 granulosa cells treated with FSH w/wo CQ. Perilipin3 (green signal) and SQSTM1 (red signal). Co-localizations of the signals are indicated beneath the image. Nuclei are stained with DAPI. Scale bars represent 20 µm. Mean ± SD, N = 3 biological replicates analyzed by one-way ANOVA, with Tukey’s test for multiple comparisons. (E) Representative confocal images of the HGrC1 granulosa cells treated with FSH w/wo CQ. Perilipin3 (green signal) and LAMP2 (red signal). Co-localizations of the signals are indicated as graphic bars beneath the image. Nuclei are stained with DAPI. Scale bars represent 20 µm. Mean ± SD, N = 3 biological replicates analyzed by one-way ANOVA, with Tukey’s test for multiple comparisons.

**Fig. S6:** Representative time-lapse images of confocal live microscopy of the luteinized granulosa cells treated with hCG (10 IU/mL) w/wo CQ (60 μM) of the patients with normal and defective luteal function. BODIPY (green signal) and lysotracker (red signal). Co-localizations of the signals over time are indicated to the right of the image. Nuclei are stained with DAPI. Scale bars represent 20 µm. Mean ± SD, N = 4 biological replicates analyzed by one-way ANOVA, with Tukey’s test for multiple comparisons.

**Fig. S7:** Our hypothetical model of the contribution of lipophagy to sex steroid hormone biosynthesis in human gonads. 1. Gonadotropin hormones accelerate autophagic flux as previously unknown function, which mediates the association of the cholesterol-laden lipid droplets with lysosome to deliver the lipid cargo within the LDs to lysosomes for degradation (lipophagy) to release free cholesterol required for steroid synthesis. 2. Illustrates the pathways previously known to be stimulated by gonadotropin hormones to enhance steroidogenesis. Confocal microscopy shows high-magnification images of the granulosa cells as a demonstration how gonadotropin stimulation promotes the association of the lipid droplets with lysosome. Scale bar 20 µm. ABCA1: ATP-binding cassette transporter; ACAT: Acyl-coenzyme A cholesterol acyltransferase also known as SOAT1, (sterol-O-acyltransferase-1); FSH: Follicle stimulating hormone; HDL: high-density lipoprotein; HMG-CoA reductase: 3-hydroxy-3-methylglutaryl-coenzyme A reductase; LDL: low-density lipoprotein; HSL: LIPE, hormone-sensitive lipase; hCG. Human chorionic gonadotropin; LH: Luteinizing hormone; NPC: Niemann-Pick type C; SR-B1, scavenger receptor type B; STAR: Steroidogenic acute regulatory protein.

**SUPPLEMENTARY MOVİES’ LEGENDS**

**Movie S1:** Confocal movie of the luteinized granulosa cells of the patients with normal luteal function. BODIPY (green signal), lysotracker (red signal).

**Movie S2:** High magnification confocal movie of the luteinized granulosa cells of the patients with normal luteal function. BODIPY (green signal), lysotracker (red signal).

**Movie S3:** Confocal movie of the luteinized granulosa cells of the patients with defective luteal function. BODIPY (green signal), lysotracker (red signal).

**Movie S4:** High magnification confocal movie of the luteinized granulosa cells of the patients with defective luteal function. BODIPY (green signal), lysotracker (red signal).

**Movie S5:** Confocal movie of the luteinized granulosa cells of the patients with normal luteal function after treatment with hCG (10 IU/mL) and chloroquine (60 μM). BODIPY (green signal), lysotracker (red signal).

**Movie S6:** Confocal movie of the luteinized granulosa cells of the patients with normal luteal function after treatment with hCG (10 IU/mL) and chloroquine (60 μM). BODIPY (green signal), lysotracker (red signal).
