# Supplementary figures and images for: Autophagy regulates sex steroid hormone synthesis through lysosomal degradation of lipid droplets in human ovary and testis
Source: Cell Death Dis. 2023 May 26;14(5):342. doi: 10.1038/s41419-023-05864-3 (PMC10220221; doi:10.1038/s41419-023-05864-3)

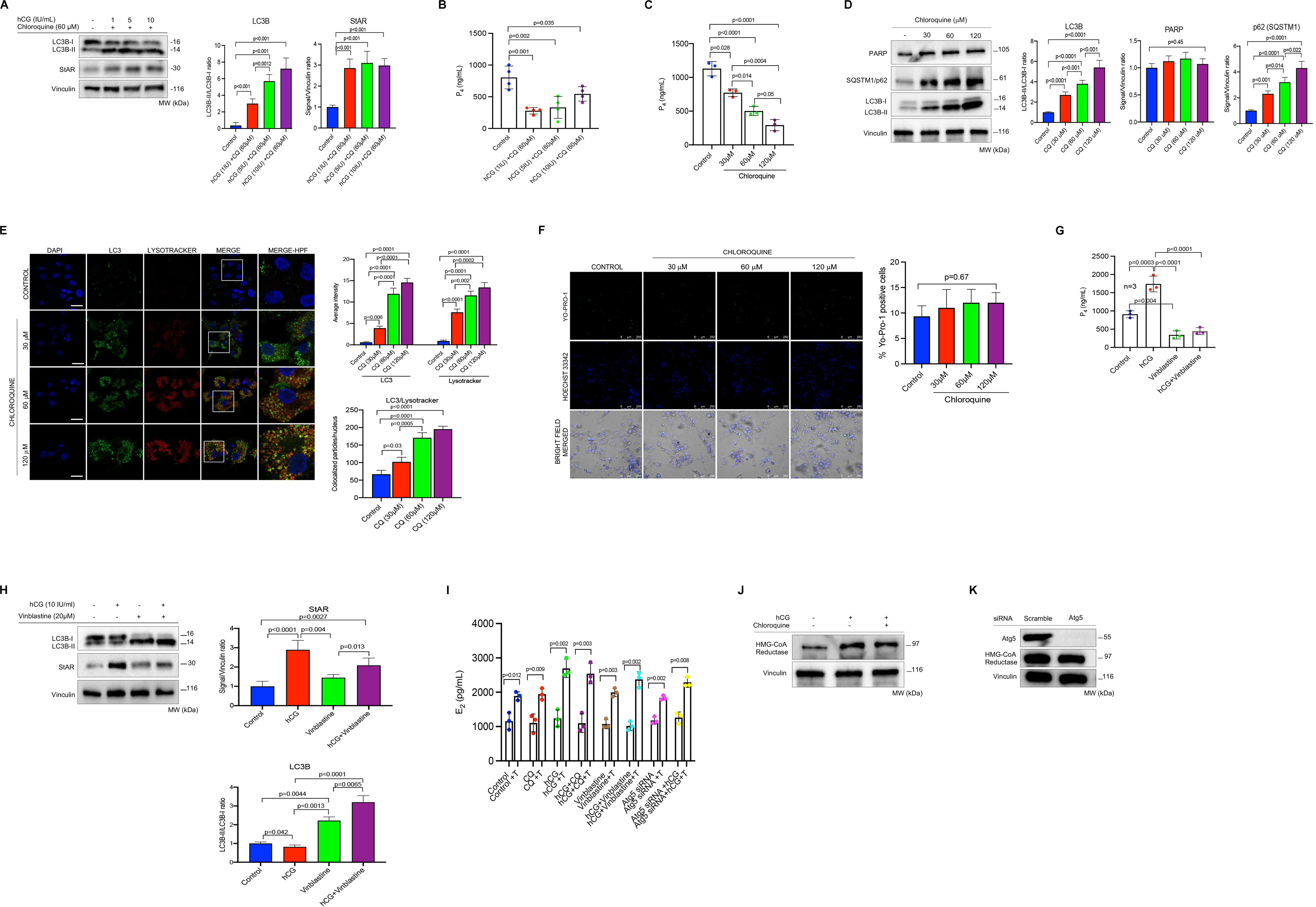

Supplement: Supplementary file 2 — Supp fig-1 [file 41419_2023_5864_MOESM2_ESM.tif]

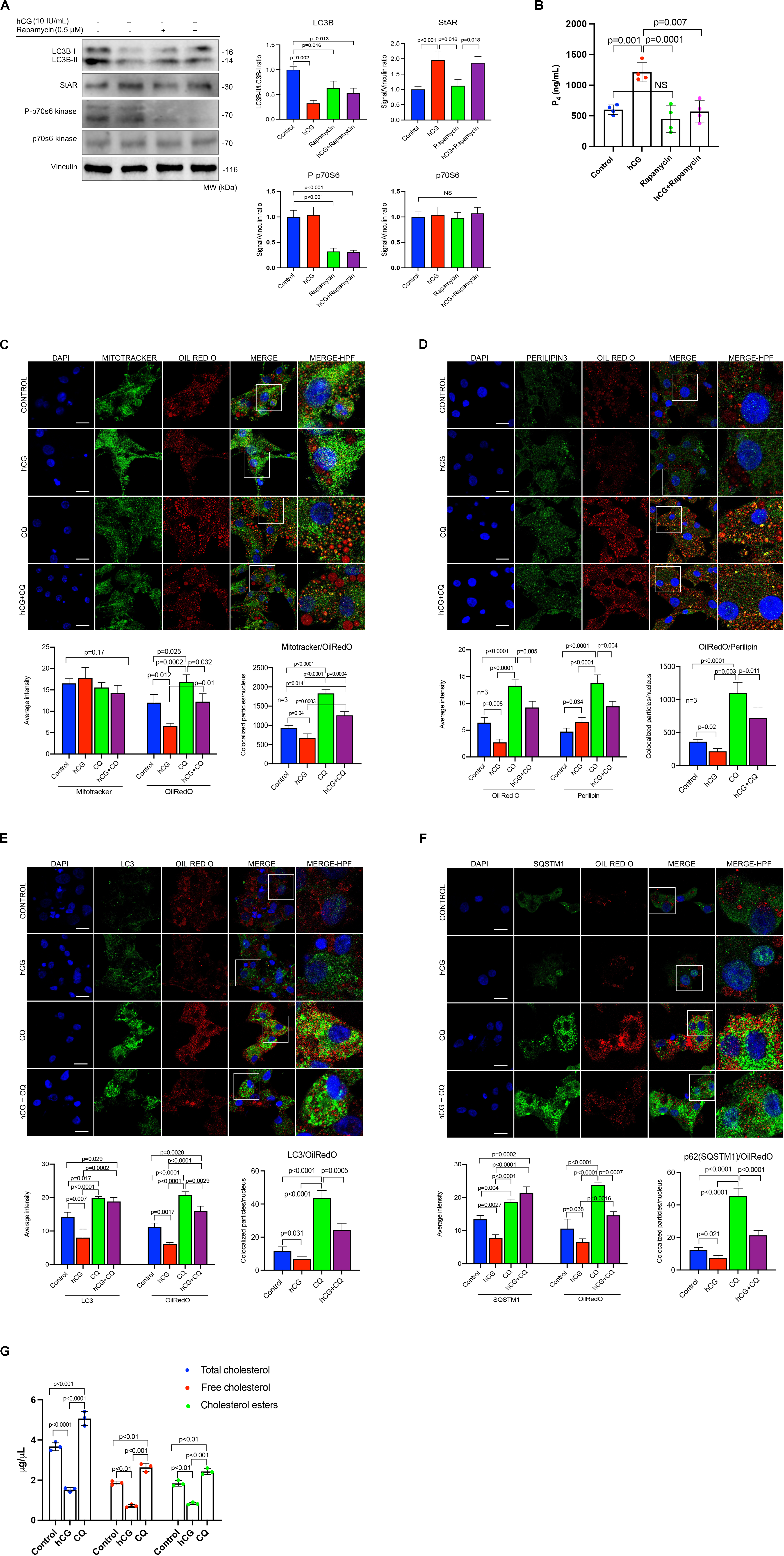

Supplement: Supplementary file 3 — Supp fig-2 [file 41419_2023_5864_MOESM3_ESM.tif]

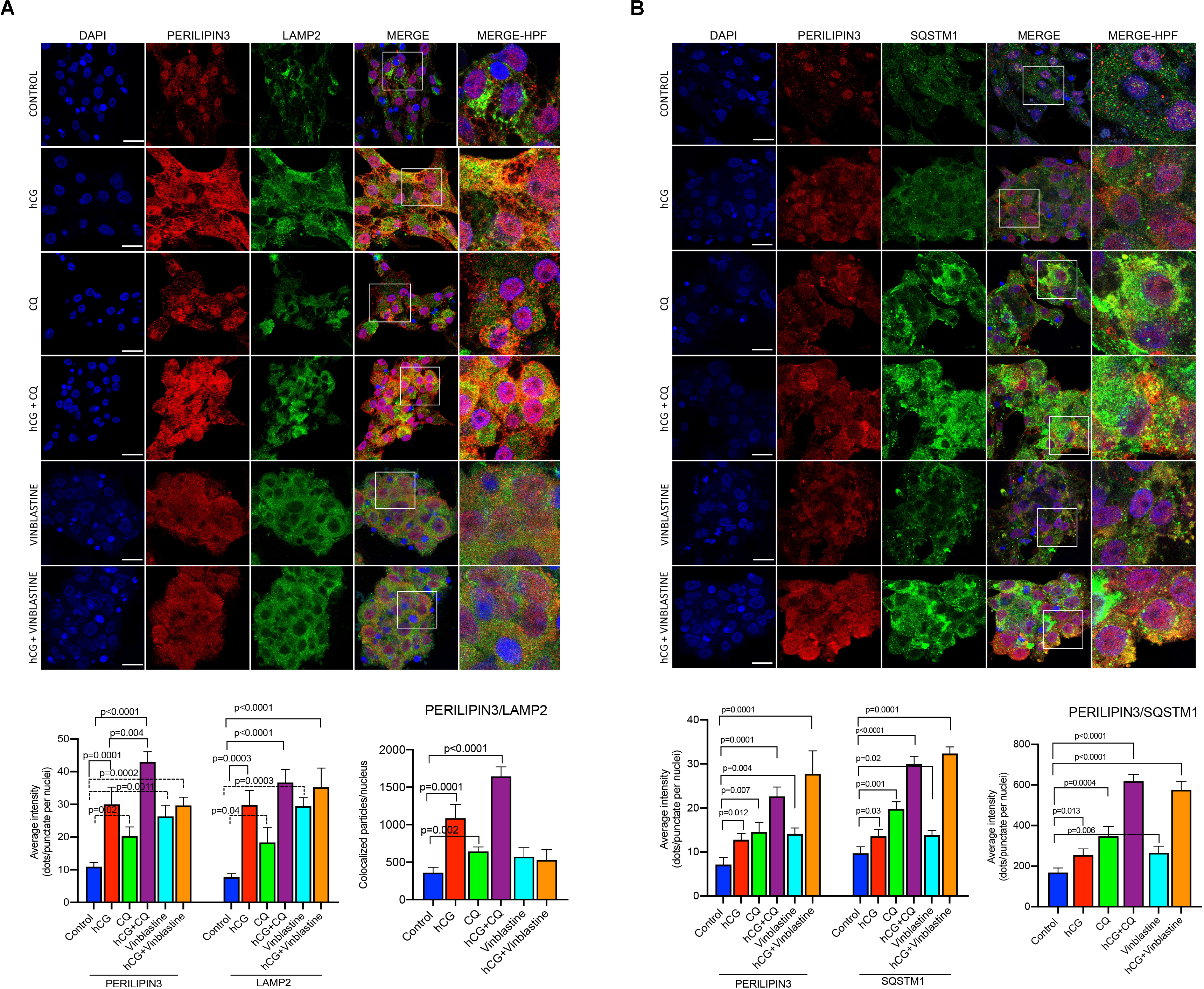

Supplement: Supplementary file 4 — Supp fig-3 [file 41419_2023_5864_MOESM4_ESM.tif]

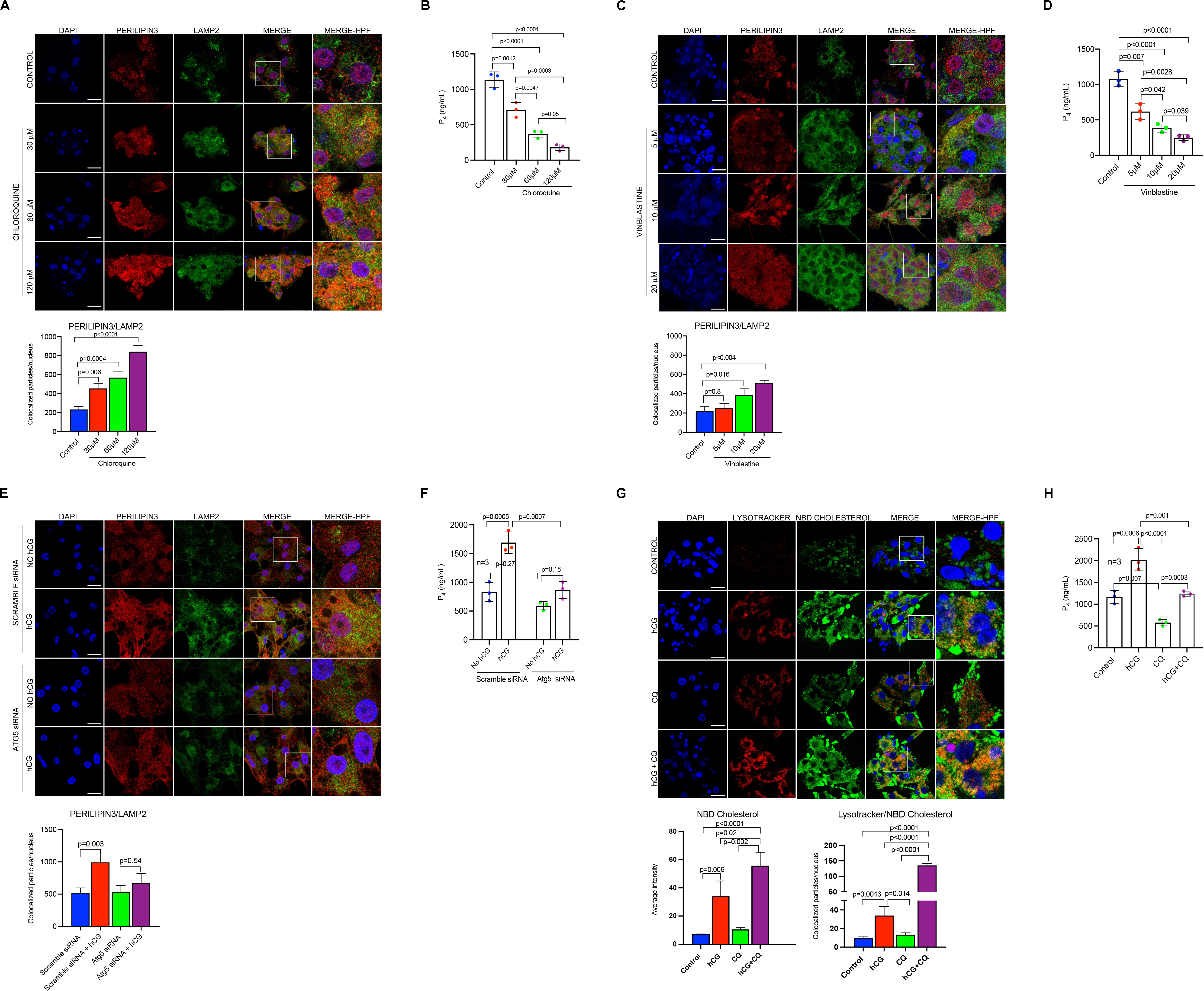

Supplement: Supplementary file 5 — Supp fig-4 [file 41419_2023_5864_MOESM5_ESM.tif]

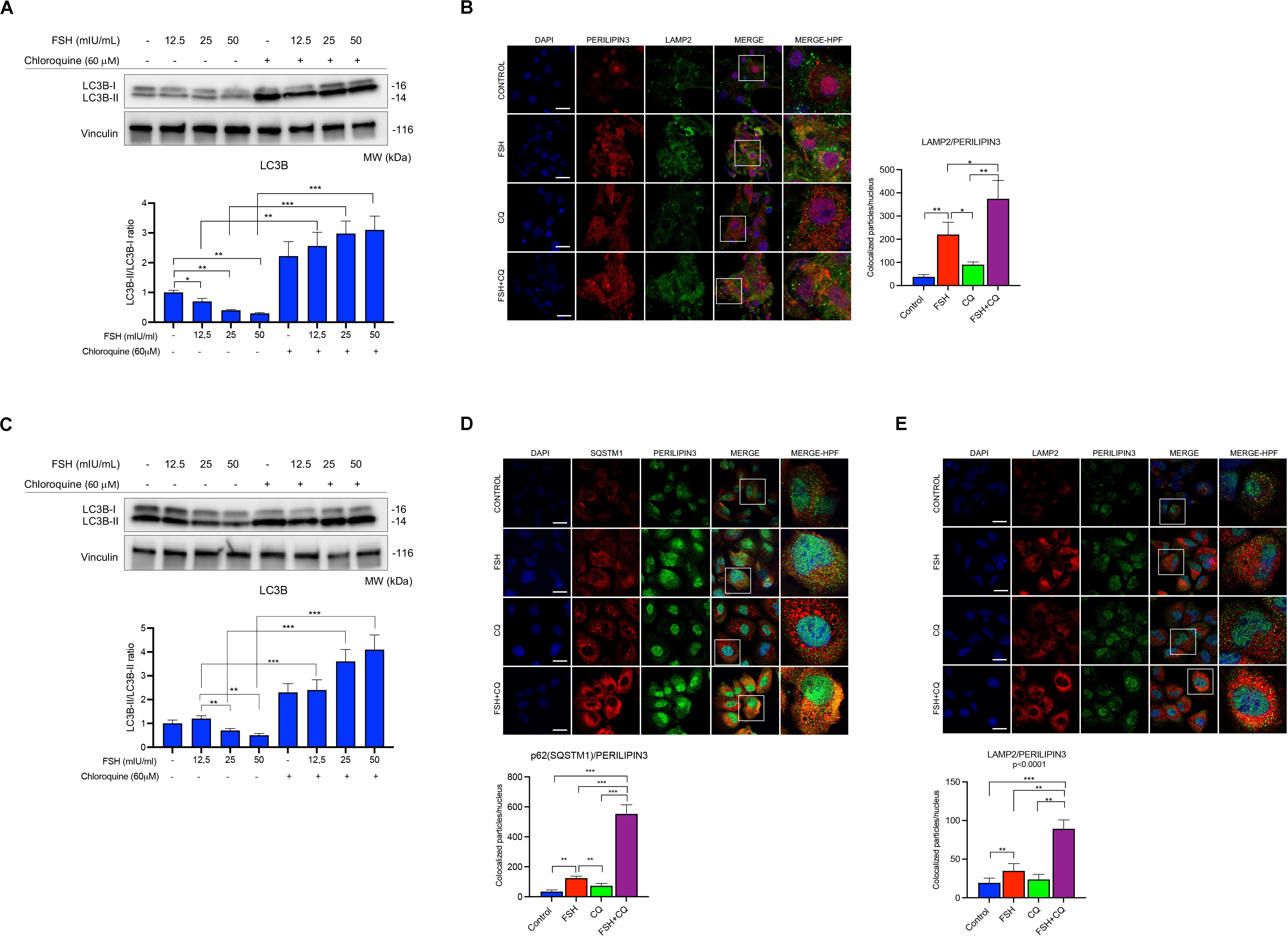

Supplement: Supplementary file 6 — Supp fig-5 [file 41419_2023_5864_MOESM6_ESM.tif]

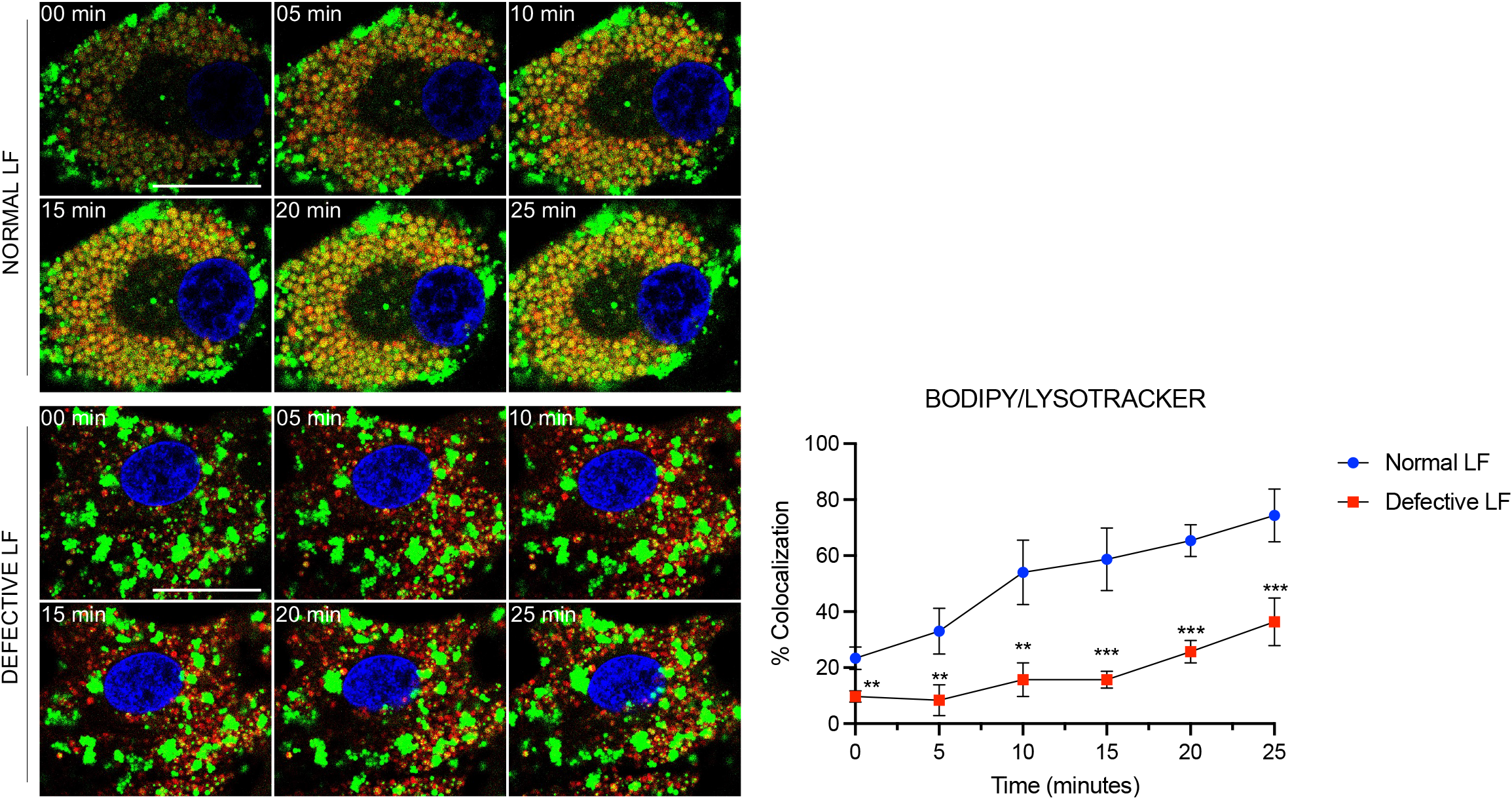

Supplement: Supplementary file 7 — Supp fig-6 [file 41419_2023_5864_MOESM7_ESM.tif]

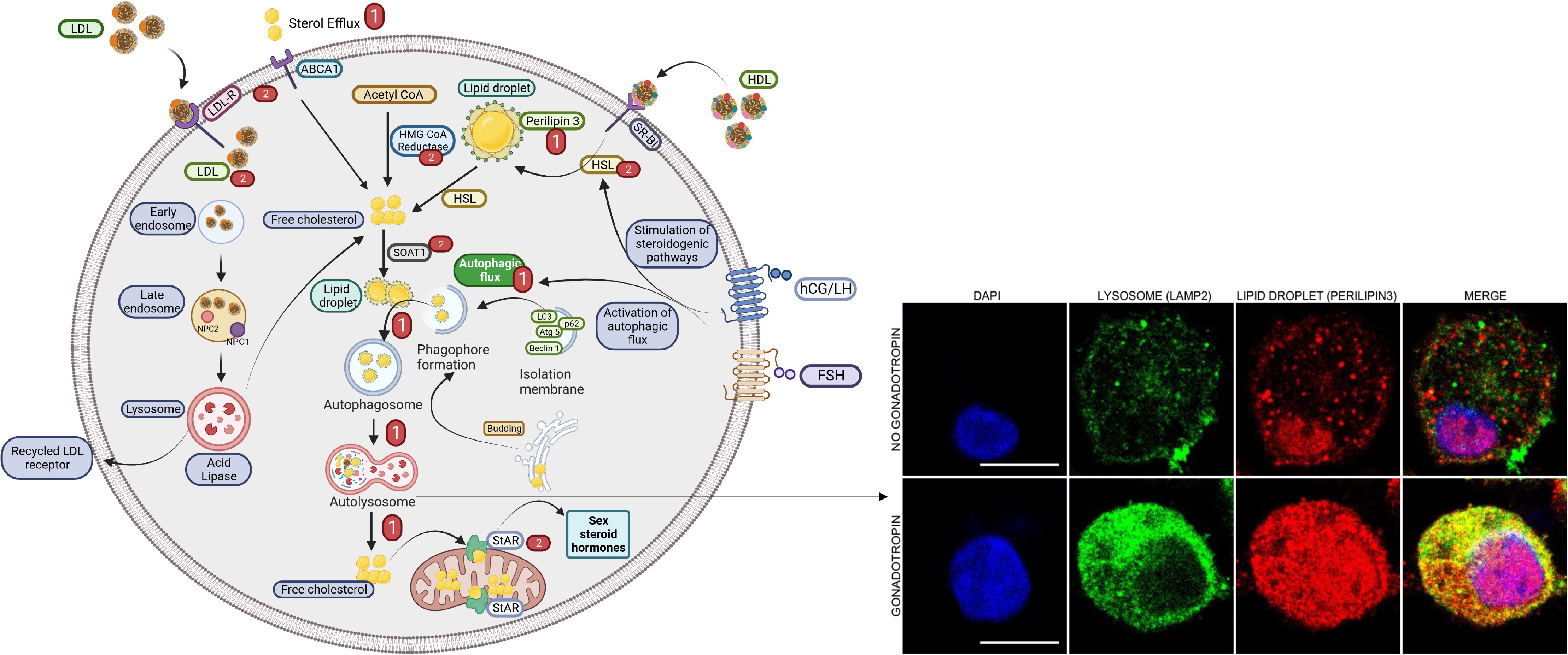

Supplement: Supplementary file 8 — Supp fig-7 [file 41419_2023_5864_MOESM8_ESM.tif]
