# Supplementary material for: Autophagy regulates sex steroid hormone synthesis through lysosomal degradation of lipid droplets in human ovary and testis
Source: Cell Death Dis. 2023 May 26;14(5):342. doi: 10.1038/s41419-023-05864-3 (PMC10220221; doi:10.1038/s41419-023-05864-3)

Fig. 1A

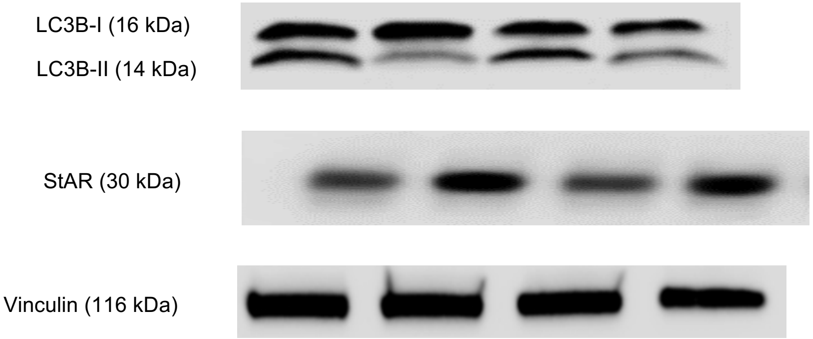
LC3B-I (16 kDa)
LC3B-II (14 kDa)
StAR (30 kDa)
Vinculin (116 kDa)

Fig. 1D

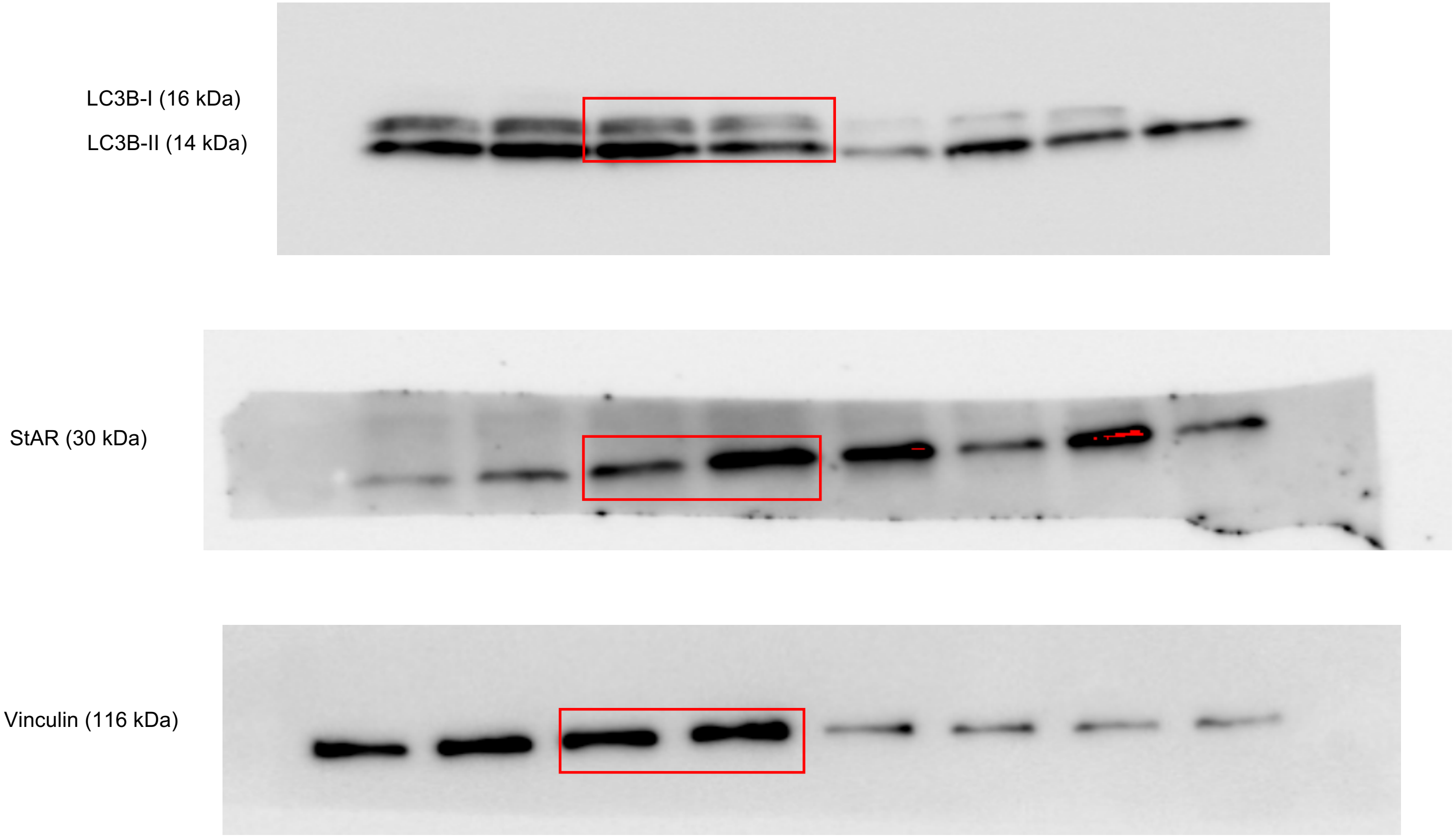
LC3B-I (16 kDa)
LC3B-II (14 kDa)
StAR (30 kDa)
Vinculin (116 kDa)

Fig. 2A

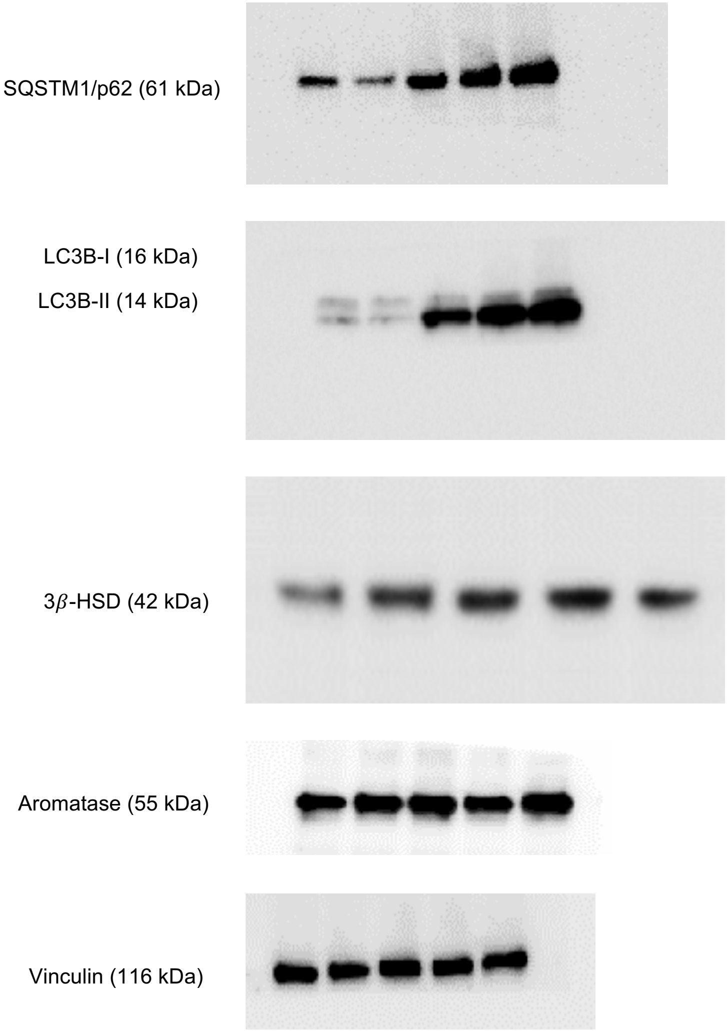
SQSTM1/p62 (61 kDa)
LC3B-I (16 kDa)
LC3B-II (14 kDa)
3β-HSD (42 kDa)
Aromatase (55 kDa)
Vinculin (116 kDa)

Fig. 2E

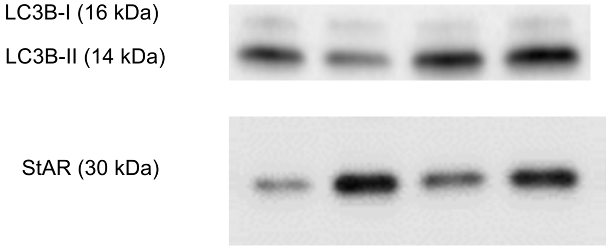
LC3B-I (16 kDa)
LC3B-II (14 kDa)
StAR (30 kDa)

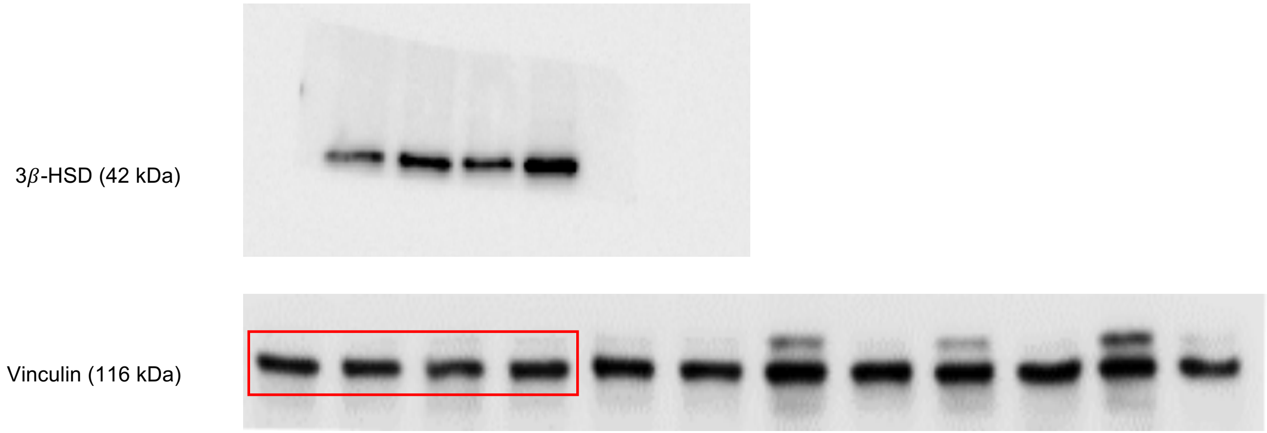
3β-HSD (42 kDa)
Vinculin (116 kDa)

Fig. 3A

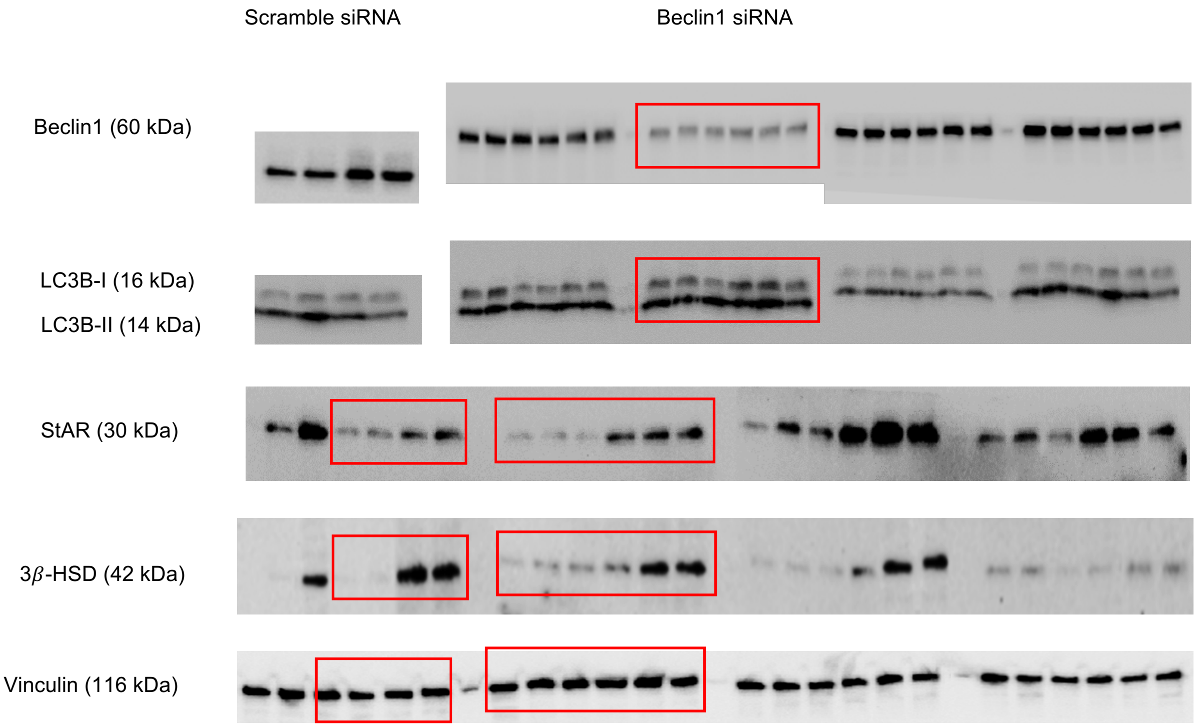
Scramble siRNA
Beclin1 siRNA
Beclin1 (60 kDa)
LC3B-I (16 kDa)
LC3B-II (14 kDa)
StAR (30 kDa)
3β-HSD (42 kDa)
Vinculin (116 kDa)

Fig. 3C

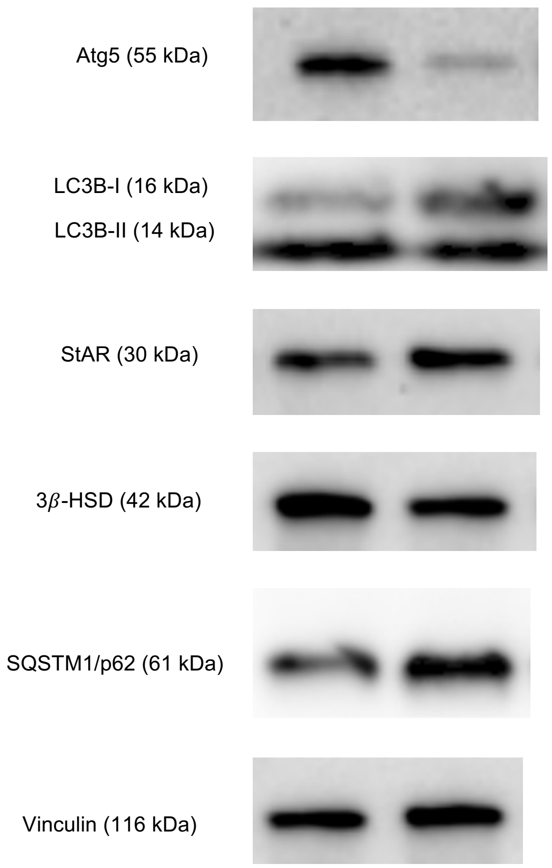
Atg5 (55 kDa)
LC3B-I (16 kDa)
LC3B-II (14 kDa)
StAR (30 kDa)
3β-HSD (42 kDa)
SQSTM1/p62 (61 kDa)
Vinculin (116 kDa)

Fig. 3E

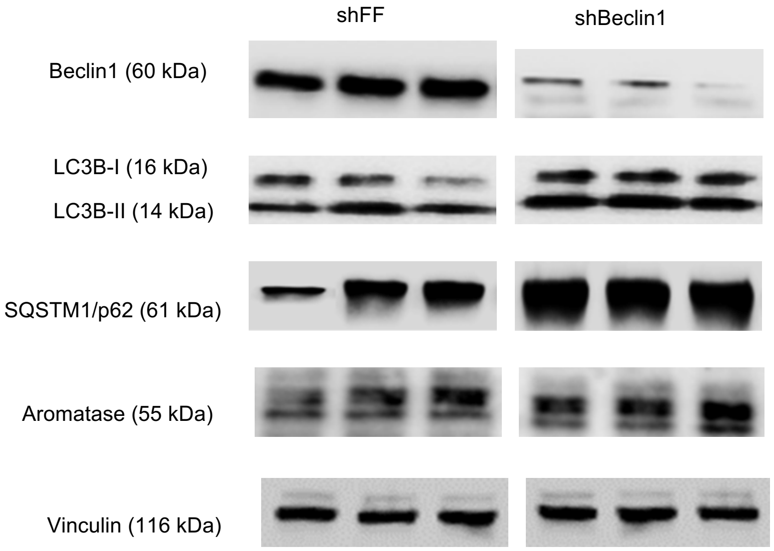
shFF
shBeclin1
Beclin1 (60 kDa)
LC3B-I (16 kDa)
LC3B-II (14 kDa)
SQSTM1/p62 (61 kDa)
Aromatase (55 kDa)
Vinculin (116 kDa)

Fig. 4B

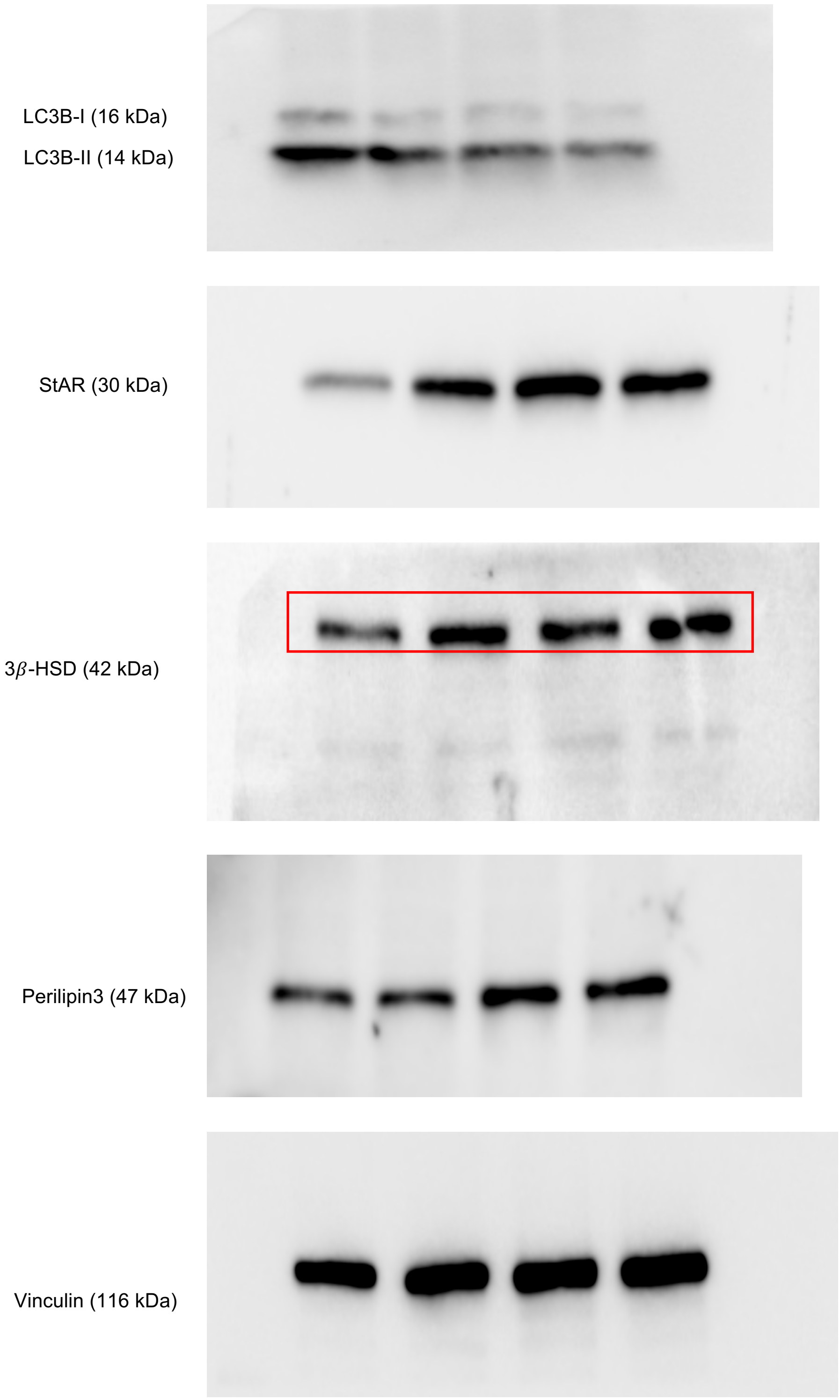
LC3B-I (16 kDa)
LC3B-II (14 kDa)
StAR (30 kDa)
3β-HSD (42 kDa)
Perilipin3 (47 kDa)
Vinculin (116 kDa)

Fig. 6C

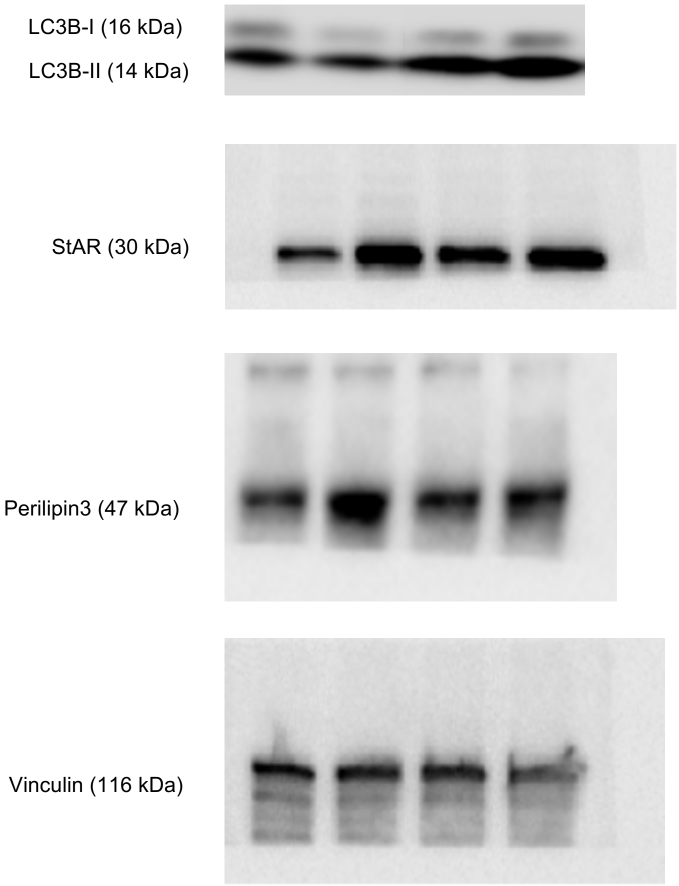
LC3B-I (16 kDa)
LC3B-II (14 kDa)
StAR (30 kDa)
Perilipin3 (47 kDa)
Vinculin (116 kDa)

Fig. 7B

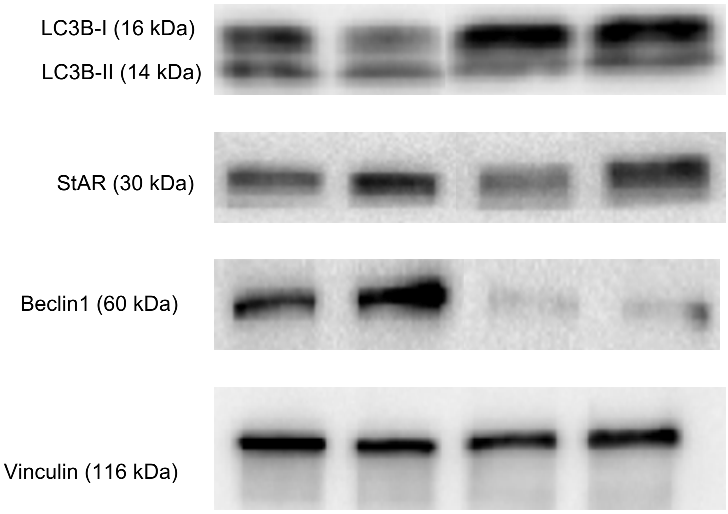
LC3B-I (16 kDa)
LC3B-II (14 kDa)
StAR (30 kDa)
Beclin1 (60 kDa)
Vinculin (116 kDa)

Fig. S1A

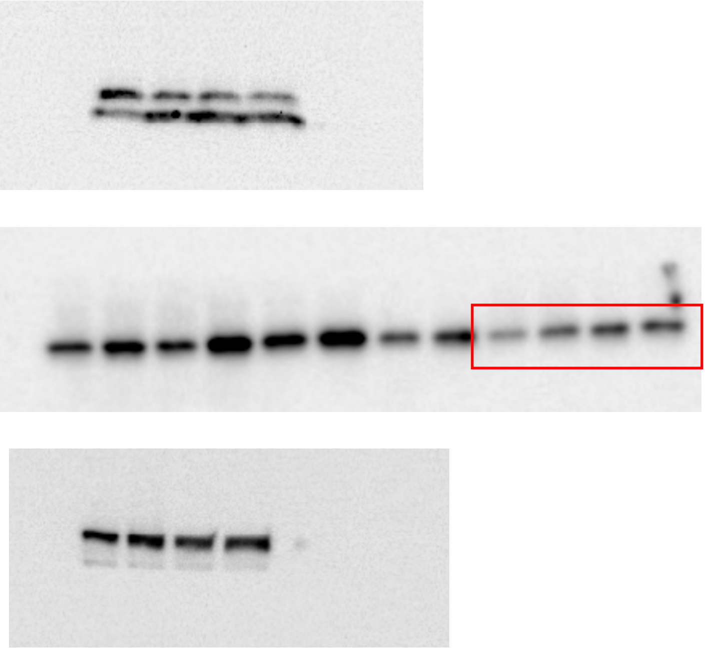

Fig. S1D

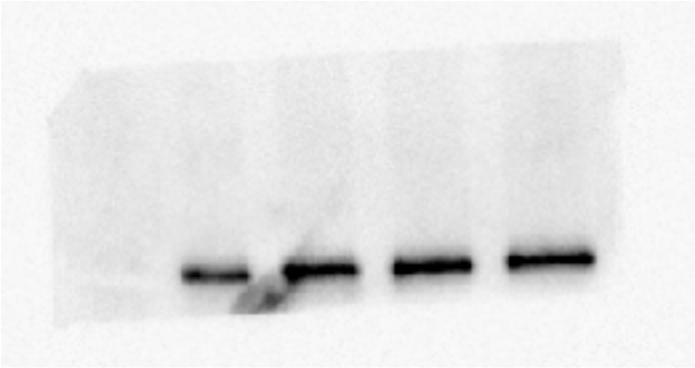

SQSTM1/p62 (61 kDa)

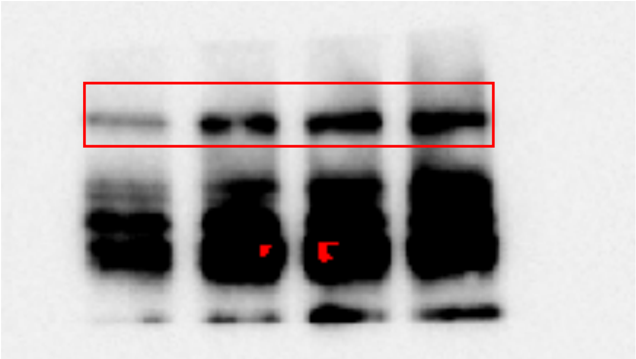

LC3B-I (16 kDa)
LC3B-II (14 kDa)

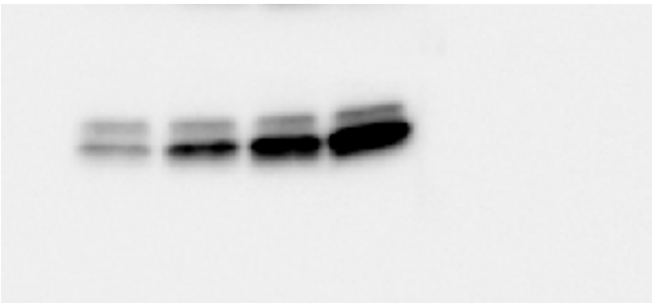

Vinculin (116 kDa)

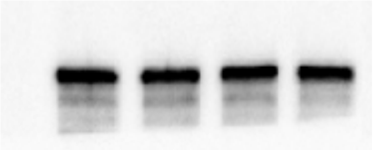

Fig. S1H

LC3B-I (16 kDa)
LC3B-II (14 kDa)

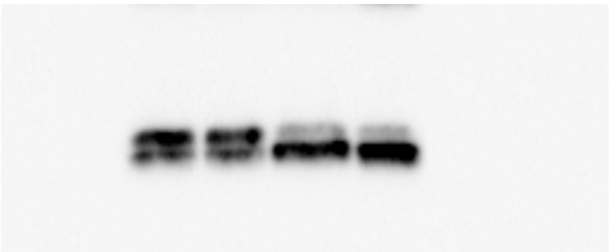

StAR (30 kDa)

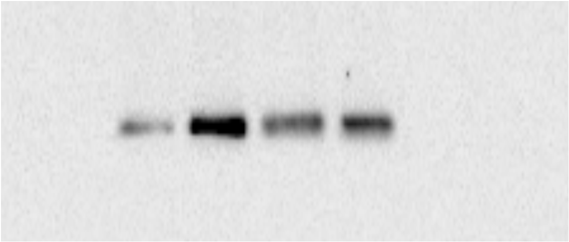

Vinculin (116 kDa)

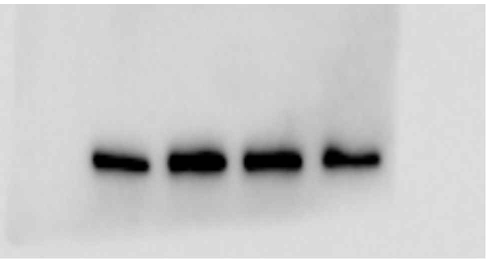

Fig. S1J

HMG-CoA
Reductase (97 kDa)

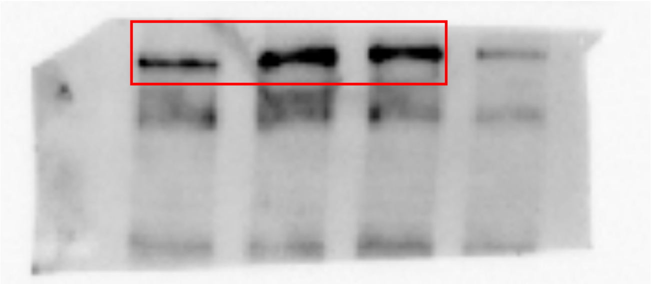

Vinculin (116 kDa)

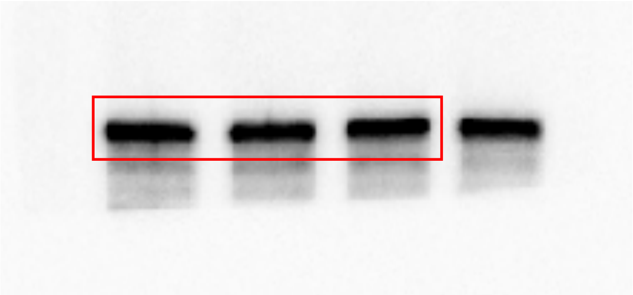

Fig. S1K

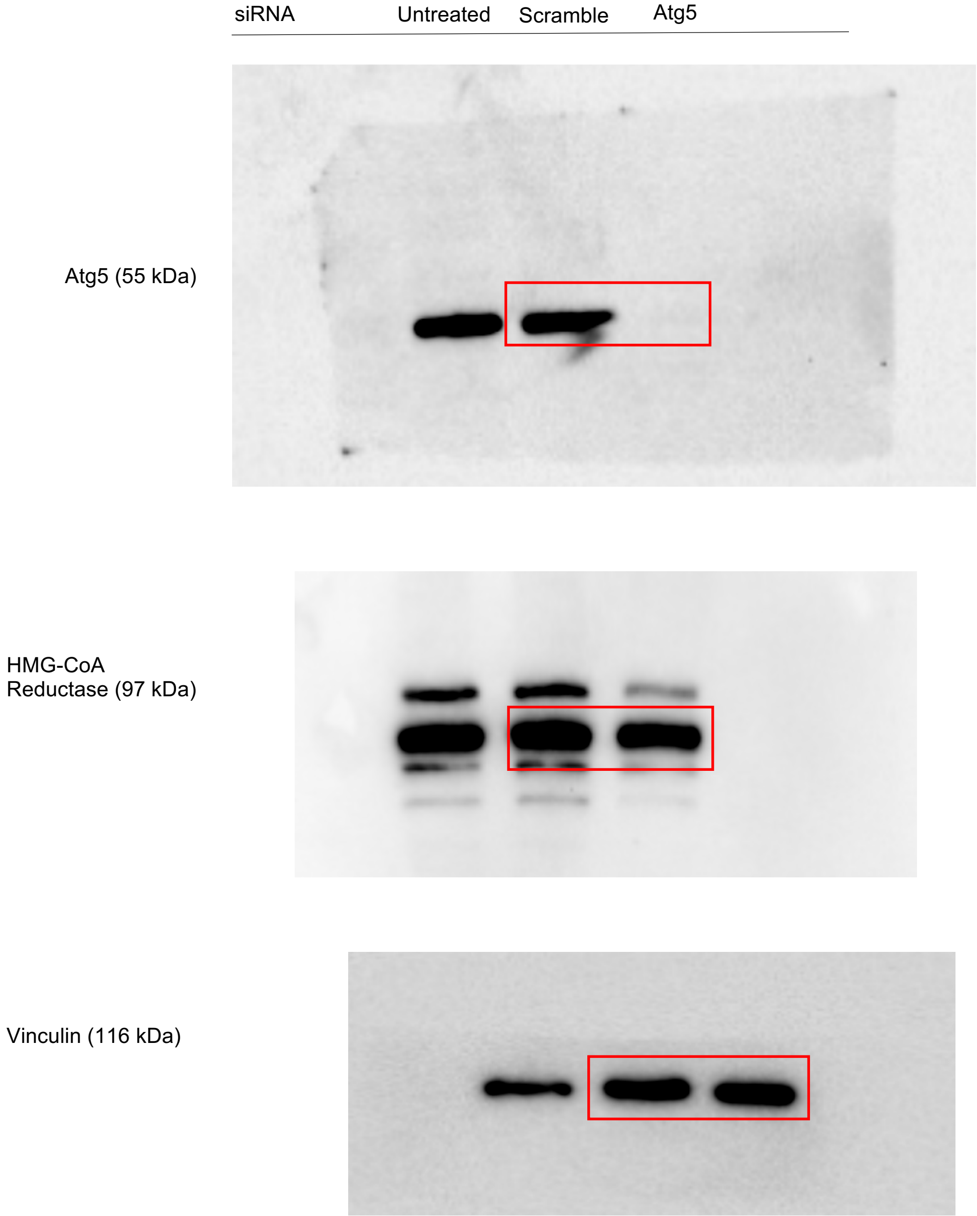
siRNA
Untreated
Scramble
Atg5
Atg5 (55 kDa)
HMG-CoA
Reductase (97 kDa)
Vinculin (116 kDa)

Fig S2A

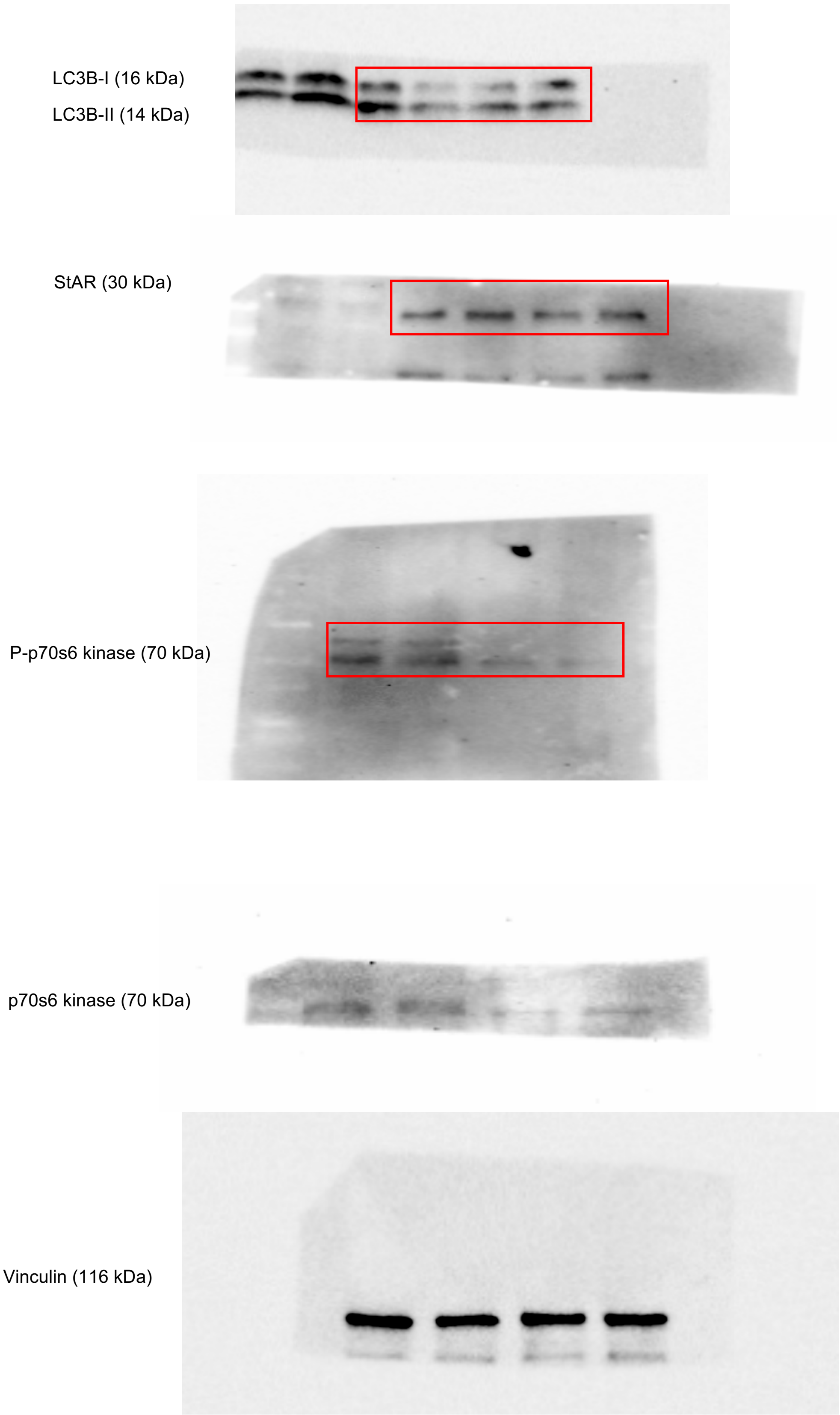

LC3B-I (16 kDa)
LC3B-II (14 kDa)
StAR (30 kDa)
P-p70s6 kinase (70 kDa)
p70s6 kinase (70 kDa)
Vinculin (116 kDa)

Fig. S5A

LC3B-I (16 kDa)

LC3B-II (14 kDa)

Vinculin (116 kDa)

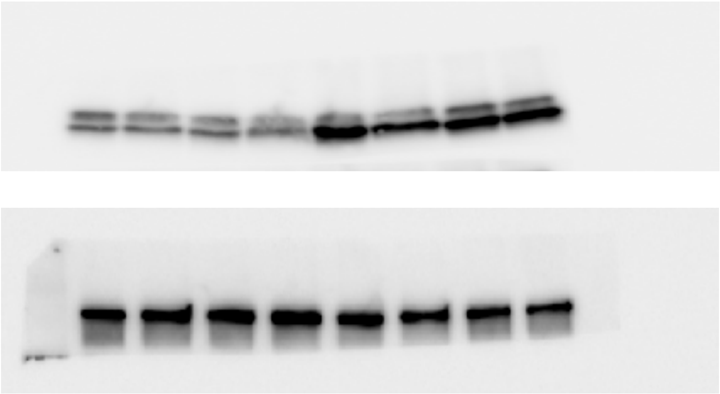

Fig. S5C

LC3B-I (16 kDa)

LC3B-II (14 kDa)

Vinculin (116 kDa)

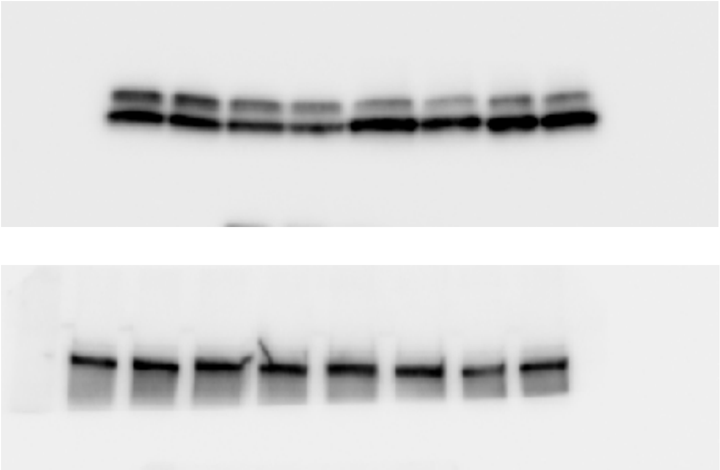

Supplement: Supplementary file 15 — Original Data File [file 41419_2023_5864_MOESM15_ESM.pdf]
